# Supplementary material for: Early Onset Ataxia with Comorbid Dystonia: Clinical, Anatomical and Biological Pathway Analysis Expose Shared Pathophysiology
Source: Diagnostics (Basel). 2020 Nov 24;10(12):997. doi: 10.3390/diagnostics10120997 (PMC7760948; doi:10.3390/diagnostics10120997)
Supplement: Supplementary file 1 [file diagnostics-10-00997-s001.zip › supplementary xml/1 Supplementary Table S1-xml.docx]

**Supplementary Table S1.** Gene mutations and Phenotypic Assessment.

| **Case** | **Diagnosis** | **Gene Mutation** | **Result Obs 1** | **Result Obs 2** | **Severity Obs 1 Prim/Sec** | **Severity Obs 2 Prim/Sec** |
| --- | --- | --- | --- | --- | --- | --- |
| **1** | cerebel cort dyspl, hypopl pons | c.4915C>T, p.(Arg1639Trp);c.5205G>C, p.(Arg1735Ser) VOUS in *RELN* | A | A | A + / None | A + / None |
| **2** | Poretti Boltzhausen | c.3919C>T, p.(Arg1307*) in *LAMA1* | AD | AD | A + / D + | A + / D + |
| **3** | Dandy Walker malformation |  | AD | AD | A + / D + | A ++ / D + |
| **4** | no |  | AD | AD | A + / D + | A +++ / D + |
| **5** | Shah Waardenburg | nonsense mutation (p.S384X) causing a premature stop codon in *SOX10* | AD | A | A + /H + | A + / None |
| **6** | CHARGE | c.1714C>T mutation in *CHD7* | AD | AD | A + / None | A ++ / None |
| **7** | pre- SCA 8 | CTG/CAG trinucleotide expansion in *ATXN8* | AD | AD | A+ / D +/- | A + / D + |
| **8** | Joubert Syndrome 23 | c.990C>T, p.(=) mutation in *KIAA0586* | AD | AD | A + / D + | A ++ / D + |
| **9** | Cediak Higashi | mutation in the lysosomal trafficking regulator, *CHS1* | AD | AD | A + / D + | A ++ / D + |
| **10** | SCA5 | c.193A>C, p.(Lys65Gln) mutation in *SPTBN2* | AD | AD | A ++ / D + | A ++ / D + |
| **11** | no |  | AD | AD | A + / D + | A +++ / D ++ |
| **12** | FA | GAA repeat expansion in intron 1 and heterozygous mutation in 3’UTR of exon 5 in *FXN* | AD | AD | A + / D + | A +++ / D + |
| **13** | AD MR 19 | c.493C>T, p.(Gln165*) mutation in *CTNNB1* | AD | AD | A + / D + | A +++ / D + |
| **14** | SCA13 | Heterozygous mutations Chr19(GRCh37):g.50826868A>G; NM_004977.2:c.1342T>C, p.(Phe448Leu) in *KCNC3* | AD | AD | A ++ / D + | A ++ / D + |
| **15** | no |  | AD | A | A + / D +/- | A ++ / None |
| **16** | MHBD-def | c.347G>A, p.(Arg116Gln) mutation in *HSD17B10* | AD | AD | A ++ / D + | A ++ / D + |
| **17** | FA | Homozygous GAA repeat expansions in intron 1 of *FXN* | AD | AD | A ++ / D + | A ++ / D + |
| **18** | HADDS | c.907C>T, p.(Arg303*) mutation in *EBF3* | AD | AD | A + / D +/- | A ++ / D + |
| **19** | FA | GAA repeat expansions in intron 1 of *FXN* | AD | A | A ++ / D +/- | A ++ / None |
| **20** | Joubert syndrome 1 | Homozygous mutation c.1762T>C, p.(Tyr588His) in *INPPE5* | AD | AD | H ++ / A +/D+ | A + / D + |
| **21** | no |  | AM | AM | A ++ / None | A ++ / M ++ |
| **22** | Northsea progr myoclonus | Homozygous mutation c.430G>T in *GOSR2* | MA | AM | M ++ / A + | A + / M ++ |
| **23** | no |  | AD | AD | A ++ / D + | A ++ / D + |
| **24** | no |  | AD | AD | A ++ / D ++ | A ++ / D + |
| **25** | no |  | AD | A | A ++ / D +/- | A ++ / None |
| **26** | EA2 | Heterozygous for mutation c. 4982G>A, p.(Arg1661His) in *CACNA1A* | A | None | A + /None | None / None |
| **27** | FA | GAA repeat expansions in intron 1 of *FXN* | A | A | A ++ / H + | A ++ / None |
| **28** | SCA19 | c.880G>T mutation in *KCND3* | A | A | A + | A ++ / None |
| **29** | EA2 | c.1050delT, p.(Phe350Leufs*3) mutation in *CACNA1A* | A | A | A + / None | A + / None |
| **30** | CAMTA1 | c.430G>T, p.(Gly144*) mutation in *CAMTA1* | AD | DA | A ++ / D + | D + / A + |
| **31** | no |  | AD | AD | A ++ / D + | A + / M ++ |
| **32** | BHC | *TITF1* gene mutation | AD | AD | A ++ / D + | A ++ / D + |
| **33** | AD, MR type 30 | c.705_708delTGAG, p.(Glu236Lysfs*52) mutation in *ZMYND11* | AD | AD | A ++ / D ++ | A + / D + |
| **34** | SCA 29 | c.4471T>G, p.(Phe1491Val) in exon 35 of *ITPR1* | A | A | A + / None | A ++ / None |
| **35** | SCA29 | c.100G>A, p.(Asp34Asn) of *ITPR1* | A | A | A + / None | A ++ / None |
| **36** | SCA29 | c.4471T>G, p.(Phe1491Val) in exon 35 of *ITPRI* | A | A | A + / None | A + / None |
| **37** | no |  | A | A | A ++ / None | A +++ / None |
| **38** | no |  | AD | DA | A ++ / D ++ | D + / A + |
| **39** | SCA5 | Heterozygous mutation c.1972C>T, p.(Arg658Trp) in *SPTBN2* | AD | AD | A ++ / M ++/ H ++ | A ++ / D + |
| **40** | FIPWE | Heterozygous mutation c.2305C>T, p.(Arg769Cys) in *ATP1A3* | DA | D | D++ / M ++ | D ++ / None |
| **41** | FIPWE | Heterozygous mutation c.2271C>A, p.(Asn757Lys) in *ATP1A3* | D | D | D +++ / None | D ++ / None |
| **42** | AVED | 487delT mutations in *TTPA* | DA | DA | D ++++ / A ++ | D +++ / A ++ |
| **43** | FA | Homozygous GAA repeat expansions in intron 1 of *FXN* | A | A | A ++ / H ++ | A +++ / H ++ |
| **44** | Niemann Pick | Compound heterozygous gene mutations c.1918G>A (+) c.3451 G>A in *NPC1* | AD | AD | A ++ / D + | A + / D + |
| **45** | AVED | 487delT mutations in *TTPA* | DA | AD | D ++ / S + | A ++ / None |
| **46** | NARP | mutation in the mitochondrial ATP synthase 6, *MTATP6* | AD | AD | A ++ / D + | A + / D + |
| **47** | AVED | 487delT mutations in *TTPA* | none | A | None / None | A + / None |
| **48** | Huntington | CAG repeat expansion in *HTT* | AD | ADM | A + / D + | A + / M + |
| **49** | AT | mutation in *ATM* | AD | AD | A +++ / D ++ | A ++ / D + |
| **50** | FA | GAA repeat expansions in intron 1 of *FXN* | AD | AD | A ++ / D + | A ++ / D + |
| **51** | Cong. FP malformation |  | AD | AD | A ++ / D ++ | A + / D + |
| **52** | KSS | mutation in the mitochondrial tRNA (leucine)-1 gene | AD | A | A ++ / D + | A +++ / None |
| **53** | FA | Homozygous GAA repeat expansions in intron 1 of *FXN* | AD | A | A ++ / H ++ | A +++ / None |
| **54** | SPG11 | mutation in spatacsin | AD | D | A + / D + | None / D + |
| **55** | Northsea progr myoclonus | Homozygous mutation c.430G>T in *GOSR2* | MAD | AM | M +++ / A + | A ++ / M +++ |
| **56** | Northsea progr myoclonus | Homozygous mutation c.430G>T in *GOSR2* | MA | AM | M +++ / A ++ | A ++ / M + |
| **57** | no |  | AD | AD | A ++ / D + | A +++ / D + |
| **58** | EA2 | c.4012G>T missense mutation in *CACNA1A* | AD | AD | A +(+) / D + | A ++ / D + |
| **59** | Joubert Syndrome 23 | two point mutations in *KIAA0586* | AD | AD | A + / D + | A + / D + |
| **60** | GOSR2 | Homozygous mutation c.430G>T in *GOSR2* | A | A | A ++ / H ++ | A ++ / H ++ |
| **61** | EA2 | c.4012G>T missense mutation in *CACNA1A* | AD | AD | A + / D + | A ++ / D + |
| **62** | AVED | mutation in *TTPA* | AD | A | A + / D + | A + / None |
| **63** | Northsea progr myoclonus | Homozygous mutation c.430G>T in *GOSR2* | ADM | AD | A ++ / D + | A + / D + |
| **64** | Northsea progr myoclonus | Homozygous mutation c.430G>T in *GOSR2* | AMD | AD | A + / D + | A + / D + |
| **65** | SjogrenLarsson | mutation in *ALDH3A2* | ASpasD | AD | A ++ / D + | A ++ / D + |
| **66** | SPG11 | mutation in spatacsin | SA | A | Spas ++ / A + | A ++ / None |
| **67** | FA | GAA repeat expansions in intron 1 of *FXN* | AD | AD | A ++ / D + | A +++ / D + |
| **68** | FA | GAA repeat expansions in intron 1 of *FXN* | AD | AC | A ++ / D + | A ++ / C + |
| **69** | FA | GAA repeat expansions in intron 1 of *FXN* | A | A | A ++ / None | A ++ / None |
| **70** | FA | GAA repeat expansions in intron 1 of *FXN* | AD | AD | A ++ / D + | A ++ / D + |
| **71** | FA | GAA repeat expansions in intron 1 of *FXN* | AD | ASpas | A ++ / D + | A +++ / Spas + |
| **72** | FA | GAA repeat expansions in intron 1 of *FXN* | AD | AD | A ++ / D + | A +++ / D + |
| **73** | CDCBM5 | Chr6(GRCh37):g.3156377C>A; NM_001069.2:c.67G>T, p.(Val23Phe) in *TUBB2A* | D | D | D ++ / None | D ++ / None |
| **74** | FIPWE | c.2305C>T, p.(Arg769Cys) mutation in *ATP1A3* | ACD | AD | A ++ / D + | A +++ / D + |
| **75** | EA2 | c.4084C>T, p.(Arg1362Trp) mutation in *CACNA1A* | AD | AD | A ++ / D + | A ++ / S ++ |
| **76** | SCA7 | CAG repeat expansion in *ATXN7* | AD | AD | A +++ / D + | A +++ / D + |
| **77** | Glut-1 def | Heterozygous mutation c.689_691delAGC, p.(Lys230_Leu231delinsMet) in *SLC2A1* | AMD | AM | A ++ / D + | A + / M + |
| **78** | no |  | AMD | AD | A ++ / D + | A + / D + |
| **79** | FA | GAA repeat expansions in intron 1 of *FXN* | AD | AD | A ++ / D + | A + / D + |
| **80** | FA | GAA repeat expansions in intron 1 of *FXN* | AD | AD | A ++ / D + | A + / D + |

Legends: Obs 1 = observer 1; Obs 2 = observer 2; A = identification of ataxia; D = identification of dystonia; In addition to ataxia and dystonia, other phenotypic features were indicated, such as M = myoclonus; Spas = identification of spasticity; C = chorea; H = hypotonia and/or muscle weakness. These features were not taken into account in the present analysis and will be addressed in a future studies. Prim = primary feature; Sec = secundary feature; + = mildly present; ++ = moderately present; +++ = severely present; pre - = early (pre-)symptomatic; cerebel cort dyspl = cerebellar cortical hypoplasia; hypopl = hypolasia; MHBD = 2-methyl-3-hydroxybutyryl- CoA-hydrogenase deficiency, HADDS = hypotonia; Ataxia and Delayed Development Syndrome; AVED = Ataxia with isolated vitamin E deficiency; NARP = neuropathy; ataxia and retinitis pigmentosa; cong malf fossa pos= congenital malformation fossa posterior; CDCBM5= cortical dysplasia, complex, with other brain malformations; FIPWE = Fever-Induced Paroxysmal Weakness and Encephalopathy.
