# Supplementary material for: Early Onset Ataxia with Comorbid Dystonia: Clinical, Anatomical and Biological Pathway Analysis Expose Shared Pathophysiology
Source: Diagnostics (Basel). 2020 Nov 24;10(12):997. doi: 10.3390/diagnostics10120997 (PMC7760948; doi:10.3390/diagnostics10120997)
Supplement: Supplementary file 1 [file diagnostics-10-00997-s001.zip › supplementary xml/10_Supplementary Table S10-xml.docx]

**Supplementary Table S10.** Enriched pathways dystonia genes.

|  | **ID** | **Name** | **P Value** | **FDR* B&H** | **FDR* B&Y** | **Bonferroni** |
| --- | --- | --- | --- | --- | --- | --- |
| 1 | GO:0045333 | cellular respiration | 7,22E-26 | 2,05E-22 | 1,75E-21 | 2,05E-22 |
| 2 | GO:0015980 | energy derivation by oxidation of organic compounds | 5,11E-21 | 7,27E-18 | 6,20E-17 | 1,45E-17 |
| 3 | GO:0055114 | oxidation-reduction process | 3,52E-18 | 3,34E-15 | 2,85E-14 | 1,00E-14 |
| 4 | GO:0006091 | generation of precursor metabolites and energy | 2,71E-16 | 1,81E-13 | 1,54E-12 | 7,72E-13 |
| 5 | GO:0022904 | respiratory electron transport chain | 3,18E-16 | 1,81E-13 | 1,54E-12 | 9,04E-13 |
| 6 | GO:0022900 | electron transport chain | 2,87E-15 | 1,18E-12 | 1,00E-11 | 8,16E-12 |
| 7 | GO:0017144 | drug metabolic process | 2,90E-15 | 1,18E-12 | 1,00E-11 | 8,25E-12 |
| 8 | GO:0033108 | mitochondrial respiratory chain complex assembly | 7,74E-15 | 2,75E-12 | 2,35E-11 | 2,20E-11 |
| 9 | GO:0007005 | mitochondrion organization | 3,33E-14 | 1,05E-11 | 8,98E-11 | 9,48E-11 |
| 10 | GO:0006163 | purine nucleotide metabolic process | 6,25E-14 | 1,78E-11 | 1,52E-10 | 1,78E-10 |
| 11 | GO:0009152 | purine ribonucleotide biosynthetic process | 7,20E-14 | 1,86E-11 | 1,59E-10 | 2,05E-10 |
| 12 | GO:0006164 | purine nucleotide biosynthetic process | 1,08E-13 | 2,55E-11 | 2,18E-10 | 3,06E-10 |
| 13 | GO:0009260 | ribonucleotide biosynthetic process | 1,70E-13 | 3,73E-11 | 3,18E-10 | 4,85E-10 |
| 14 | GO:0072522 | purine-containing compound biosynthetic process | 2,35E-13 | 4,75E-11 | 4,05E-10 | 6,68E-10 |
| 15 | GO:0046390 | ribose phosphate biosynthetic process | 2,50E-13 | 4,75E-11 | 4,05E-10 | 7,12E-10 |
| 16 | GO:0072521 | purine-containing compound metabolic process | 2,69E-13 | 4,79E-11 | 4,08E-10 | 7,66E-10 |
| 17 | GO:0009150 | purine ribonucleotide metabolic process | 3,85E-13 | 6,45E-11 | 5,50E-10 | 1,10E-09 |
| 18 | GO:0042775 | mitochondrial ATP synthesis coupled electron transport | 6,19E-13 | 9,79E-11 | 8,35E-10 | 1,76E-09 |
| 19 | GO:0042773 | ATP synthesis coupled electron transport | 7,24E-13 | 1,05E-10 | 8,97E-10 | 2,06E-09 |
| 20 | GO:0009259 | ribonucleotide metabolic process | 7,39E-13 | 1,05E-10 | 8,97E-10 | 2,10E-09 |
| 21 | GO:0019693 | ribose phosphate metabolic process | 1,08E-12 | 1,47E-10 | 1,25E-09 | 3,08E-09 |
| 22 | GO:1901293 | nucleoside phosphate biosynthetic process | 2,09E-12 | 2,70E-10 | 2,30E-09 | 5,94E-09 |
| 23 | GO:0010257 | NADH dehydrogenase complex assembly | 2,83E-12 | 3,35E-10 | 2,86E-09 | 8,04E-09 |
| 24 | GO:0032981 | mitochondrial respiratory chain complex I assembly | 2,83E-12 | 3,35E-10 | 2,86E-09 | 8,04E-09 |
| 25 | GO:0009117 | nucleotide metabolic process | 6,03E-12 | 6,87E-10 | 5,86E-09 | 1,72E-08 |
| 26 | GO:0006753 | nucleoside phosphate metabolic process | 8,05E-12 | 8,82E-10 | 7,52E-09 | 2,29E-08 |
| 27 | GO:0006119 | oxidative phosphorylation | 1,52E-11 | 1,60E-09 | 1,37E-08 | 4,32E-08 |
| 28 | GO:0015985 | energy coupled proton transport, down electrochemical gradient | 2,08E-11 | 2,05E-09 | 1,74E-08 | 5,93E-08 |
| 29 | GO:0015986 | ATP synthesis coupled proton transport | 2,08E-11 | 2,05E-09 | 1,74E-08 | 5,93E-08 |
| 30 | GO:0006120 | mitochondrial electron transport, NADH to ubiquinone | 2,43E-11 | 2,30E-09 | 1,97E-08 | 6,91E-08 |
| 31 | GO:0009165 | nucleotide biosynthetic process | 2,80E-11 | 2,57E-09 | 2,20E-08 | 7,98E-08 |
| 32 | GO:0009141 | nucleoside triphosphate metabolic process | 5,34E-11 | 4,75E-09 | 4,05E-08 | 1,52E-07 |
| 33 | GO:0051186 | cofactor metabolic process | 5,91E-11 | 5,10E-09 | 4,35E-08 | 1,68E-07 |
| 34 | GO:0009142 | nucleoside triphosphate biosynthetic process | 1,10E-10 | 9,17E-09 | 7,82E-08 | 3,12E-07 |
| 35 | GO:0055086 | nucleobase-containing small molecule metabolic process | 1,50E-10 | 1,22E-08 | 1,04E-07 | 4,26E-07 |
| 36 | GO:0019637 | organophosphate metabolic process | 1,65E-10 | 1,31E-08 | 1,12E-07 | 4,71E-07 |
| 37 | GO:0009144 | purine nucleoside triphosphate metabolic process | 1,76E-10 | 1,36E-08 | 1,16E-07 | 5,02E-07 |
| 38 | GO:0006754 | ATP biosynthetic process | 2,30E-10 | 1,72E-08 | 1,47E-07 | 6,54E-07 |
| 39 | GO:0009060 | aerobic respiration | 2,58E-10 | 1,88E-08 | 1,60E-07 | 7,33E-07 |
| 40 | GO:0090407 | organophosphate biosynthetic process | 4,66E-10 | 3,31E-08 | 2,83E-07 | 1,33E-06 |
| 41 | GO:0006732 | coenzyme metabolic process | 5,52E-10 | 3,83E-08 | 3,27E-07 | 1,57E-06 |
| 42 | GO:0009206 | purine ribonucleoside triphosphate biosynthetic process | 6,41E-10 | 4,34E-08 | 3,71E-07 | 1,82E-06 |
| 43 | GO:0009145 | purine nucleoside triphosphate biosynthetic process | 6,96E-10 | 4,61E-08 | 3,93E-07 | 1,98E-06 |
| 44 | GO:0009201 | ribonucleoside triphosphate biosynthetic process | 1,04E-09 | 6,75E-08 | 5,76E-07 | 2,97E-06 |
| 45 | GO:0009205 | purine ribonucleoside triphosphate metabolic process | 1,81E-09 | 1,14E-07 | 9,75E-07 | 5,15E-06 |
| 46 | GO:0009199 | ribonucleoside triphosphate metabolic process | 3,07E-09 | 1,90E-07 | 1,62E-06 | 8,73E-06 |
| 47 | GO:1901135 | carbohydrate derivative metabolic process | 1,11E-08 | 6,74E-07 | 5,75E-06 | 3,17E-05 |
| 48 | GO:1901135 | proton transmembrane transport | 1,19E-08 | 7,05E-07 | 6,01E-06 | 3,38E-05 |

| 49 | GO:0046034 | ATP metabolic process | 1,26E-08 | 7,31E-07 | 6,23E-06 | 3,58E-05 |
| --- | --- | --- | --- | --- | --- | --- |
| 50 | GO:0006811 | ion transport | 1,86E-08 | 1,06E-06 | 9,02E-06 | 5,29E-05 |
| 51 | GO:0051188 | cofactor biosynthetic process | 2,00E-08 | 1,12E-06 | 9,54E-06 | 5,70E-05 |
| 52 | GO:1901137 | carbohydrate derivative biosynthetic process | 4,42E-08 | 2,42E-06 | 2,07E-05 | 1,26E-04 |
| 53 | GO:0019752 | carboxylic acid metabolic process | 1,15E-07 | 6,20E-06 | 5,29E-05 | 3,29E-04 |
| 54 | GO:0009108 | coenzyme biosynthetic process | 1,92E-07 | 1,01E-05 | 8,65E-05 | 5,47E-04 |
| 55 | GO:0034220 | ion transmembrane transport | 3,03E-07 | 1,57E-05 | 1,34E-04 | 8,63E-04 |
| 56 | GO:0046146 | tetrahydrobiopterin metabolic process | 7,29E-07 | 3,70E-05 | 3,16E-04 | 2,07E-03 |
| 57 | GO:0043436 | oxoacid metabolic process | 7,84E-07 | 3,91E-05 | 3,34E-04 | 2,23E-03 |
| 58 | GO:0006812 | cation transport | 8,50E-07 | 4,17E-05 | 3,56E-04 | 2,42E-03 |
| 59 | GO:0006082 | organic acid metabolic process | 1,14E-06 | 5,50E-05 | 4,69E-04 | 3,24E-03 |
| 60 | GO:0098662 | inorganic cation transmembrane transport | 2,44E-06 | 1,16E-04 | 9,88E-04 | 6,95E-03 |
| 61 | GO:0098660 | inorganic ion transmembrane transport | 2,61E-06 | 1,22E-04 | 1,04E-03 | 7,44E-03 |
| 62 | GO:0098655 | cation transmembrane transport | 7,18E-06 | 3,30E-04 | 2,81E-03 | 2,04E-02 |
| 63 | GO:0015672 | monovalent inorganic cation transport | 8,64E-05 | 3,90E-03 | 3,33E-02 | 2,46E-01 |
| 64 | GO:0055085 | transmembrane transport | 9,00E-05 | 4,00E-03 | 3,41E-02 | 2,56E-01 |
| 65 | GO:0006729 | tetrahydrobiopterin biosynthetic process | 1,10E-04 | 4,74E-03 | 4,05E-02 | 3,13E-01 |
| 66 | GO:0061732 | mitochondrial acetyl-CoA biosynthetic process from pyruvate | 1,10E-04 | 4,74E-03 | 4,05E-02 | 3,13E-01 |
| 67 | GO:1901615 | organic hydroxy compound metabolic process | 1,98E-04 | 8,42E-03 | 7,18E-02 | 5,64E-01 |
| 68 | GO:0009712 | catechol-containing compound metabolic process | 3,48E-04 | 1,43E-02 | 1,22E-01 | 9,90E-01 |
| 69 | GO:0006584 | catecholamine metabolic process | 3,48E-04 | 1,43E-02 | 1,22E-01 | 9,90E-01 |
| 70 | GO:0042558 | pteridine-containing compound metabolic process | 5,22E-04 | 2,12E-02 | 1,81E-01 | 1,49E+00 |
| 71 | GO:0009072 | aromatic amino acid family metabolic process | 9,06E-04 | 3,58E-02 | 3,06E-01 | 2,58E+00 |
| 72 | GO:0042417 | dopamine metabolic process | 9,06E-04 | 3,58E-02 | 3,06E-01 | 2,58E+00 |
| 73 | GO:0008535 | respiratory chain complex IV assembly | 1,10E-03 | 4,30E-02 | 3,67E-01 | 3,14E+00 |

| 74 | GO:0034311 | diol metabolic process | 1,45E-03 | 5,42E- 02 | 4,62E- 01 | 4,12E+00 |
| --- | --- | --- | --- | --- | --- | --- |
| 75 | GO:0042423 | catecholamine biosynthetic process | 1,45E-03 | 5,42E- 02 | 4,62E- 01 | 4,12E+00 |
| 76 | GO:0009713 | catechol-containing compound biosynthetic process | 1,45E-03 | 5,42E- 02 | 4,62E- 01 | 4,12E+00 |
| 77 | GO:0042416 | dopamine biosynthetic process | 2,48E-03 | 9,17E- 02 | 7,82E- 01 | 7,06E+00 |
| 78 | GO:0034622 | cellular protein-containing complex assembly | 2,81E-03 | 1,03E- 01 | 8,75E- 01 | 8,00E+00 |
| 79 | GO:0018958 | phenol-containing compound metabolic process | 3,59E-03 | 1,29E- 01 | 1,10E+ 00 | 1,02E+01 |
| 80 | GO:0042559 | pteridine-containing compound biosynthetic process | 5,75E-03 | 2,04E- 01 | 1,74E+ 00 | 1,64E+01 |
| 81 | GO:0006086 | acetyl-CoA biosynthetic process from pyruvate | 8,33E-03 | 2,93E- 01 | 2,50E+ 00 | 2,37E+01 |
| 82 | GO:0017004 | cytochrome complex assembly | 1,06E-02 | 3,69E- 01 | 3,15E+ 00 | 3,02E+01 |
| 83 | GO:0006099 | tricarboxylic acid cycle | 1,28E-02 | 4,38E- 01 | 3,74E+ 00 | 3,64E+01 |
| 84 | GO:0034312 | diol biosynthetic process | 2,91E-02 | 9,65E- 01 | 8,23E+ 00 | 8,29E+01 |
| 85 | GO:0033866 | nucleoside bisphosphate biosynthetic process | 2,95E-02 | 9,65E- 01 | 8,23E+ 00 | 8,40E+01 |
| 86 | GO:0034033 | purine nucleoside bisphosphate biosynthetic process | 2,95E-02 | 9,65E- 01 | 8,23E+  00 | 8,40E+01 |
| 87 | GO:0034030 | ribonucleoside bisphosphate biosynthetic process | 2,95E-02 | 9,65E- 01 | 8,23E+ 00 | 8,40E+01 |
| 88 | GO:0034032 | purine nucleoside bisphosphate metabolic process | 3,05E-02 | 9,65E- 01 | 8,23E+  00 | 8,69E+01 |
| 89 | GO:0033875 | ribonucleoside bisphosphate metabolic process | 3,05E-02 | 9,65E- 01 | 8,23E+ 00 | 8,69E+01 |
| 90 | GO:0033865 | nucleoside bisphosphate metabolic process | 3,05E-02 | 9,65E- 01 | 8,23E+ 00 | 8,69E+01 |
| 91 | GO:0006085 | acetyl-CoA biosynthetic process | 4,91E-02 | 1,54E+ 00 | 1,31E+ 01 | 1,40E+02 |
| 92 | GO:0035383 | thioester metabolic process | 5,69E-02 | 1,74E+ 00 | 1,48E+ 01 | 1,62E+02 |
| 93 | GO:0006637 | acyl-CoA metabolic process | 5,69E-02 | 1,74E+ 00 | 1,48E+ 01 | 1,62E+02 |
| 94 | GO:1901605 | alpha-amino acid metabolic process | 9,07E-02 | 2,75E+ 00 | 2,34E+ 01 | 2,58E+02 |
| 95 | GO:0046189 | phenol-containing compound biosynthetic process | 1,04E-01 | 3,13E+ 00 | 2,67E+ 01 | 2,97E+02 |
| 96 | GO:0032787 | monocarboxylic acid metabolic process | 1,30E-01 | 3,84E+ 00 | 3,28E+ 01 | 3,69E+02 |
| 97 | GO:0006520 | cellular amino acid metabolic process | 1,35E-01 | 3,97E+ 00 | 3,39E+ 01 | 3,85E+02 |
| 98 | GO:0071616 | acyl-CoA biosynthetic process | 1,89E-01 | 5,44E+ 00 | 4,64E+ 01 | 5,39E+02 |

| 99 | GO:0035384 | thioester biosynthetic process | 1,89E-01 | 5,44E+ 00 | 4,64E+ 01 | 5,39E+02 |
| --- | --- | --- | --- | --- | --- | --- |
| 100 | GO:0006558 | L-phenylalanine metabolic process | 2,14E-01 | 6,02E+ 00 | 5,14E+ 01 | 6,08E+02 |
| 101 | GO:1902221 | erythrose 4-phosphate/phosphoenolpyruvate family amino acid metabolic process | 2,14E-01 | 6,02E+ 00 | 5,14E+ 01 | 6,08E+02 |
| 102 | GO:0010035 | response to inorganic substance | 2,52E-01 | 7,03E+ 00 | 5,99E+ 01 | 7,17E+02 |
| 103 | GO:0007626 | locomotory behavior | 2,93E-01 | 8,09E+ 00 | 6,90E+ 01 | 8,34E+02 |
| 104 | GO:0071287 | cellular response to manganese ion | 5,83E-01 | 1,60E+ 01 | 1,36E+ 02 | 1,66E+03 |
| 105 | GO:0001505 | regulation of neurotransmitter levels | 6,67E-01 | 1,81E+ 01 | 1,54E+ 02 | 1,90E+03 |
| 106 | GO:1901617 | organic hydroxy compound biosynthetic process | 7,66E-01 | 2,06E+ 01 | 1,75E+ 02 | 2,18E+03 |
| 107 | GO:0097164 | ammonium ion metabolic process | 9,27E-01 | 2,47E+ 01 | 2,10E+ 02 | 2,64E+03 |
| 108 | GO:0006084 | acetyl-CoA metabolic process | 1,15E+00 | 3,03E+ 01 | 2,58E+ 02 | 3,27E+03 |
| 109 | GO:0001963 | synaptic transmission, dopaminergic | 1,16E+00 | 3,03E+ 01 | 2,59E+ 02 | 3,31E+03 |
| 110 | GO:0050884 | neuromuscular process controlling posture | 1,29E+00 | 3,35E+ 01 | 2,86E+ 02 | 3,68E+03 |
| 111 | GO:1903146 | regulation of autophagy of mitochondrion | 1,66E+00 | 4,26E+ 01 | 3,63E+ 02 | 4,73E+03 |
| 112 | GO:0061726 | mitochondrion disassembly | 1,86E+00 | 4,67E+ 01 | 3,99E+ 02 | 5,28E+03 |
| 113 | GO:0000422 | autophagy of mitochondrion | 1,86E+00 | 4,67E+ 01 | 3,99E+ 02 | 5,28E+03 |
| 114 | GO:1903207 | regulation of hydrogen peroxide-induced neuron death | 1,95E+00 | 4,78E+ 01 | 4,08E+ 02 | 5,54E+03 |
| 115 | GO:1903208 | negative regulation of hydrogen peroxide-induced neuron death | 1,95E+00 | 4,78E+ 01 | 4,08E+ 02 | 5,54E+03 |
| 116 | GO:0036476 | neuron death in response to hydrogen peroxide | 1,95E+00 | 4,78E+ 01 | 4,08E+ 02 | 5,54E+03 |
| 117 | GO:0033617 | mitochondrial respiratory chain complex IV assembly | 2,03E+00 | 4,90E+01 | 4,18E+ 02 | 5,79E+03 |
| 118 | GO:1903599 | positive regulation of autophagy of mitochondrion | 2,03E+00 | 4,90E+ 01 | 4,18E+ 02 | 5,79E+03 |
| 119 | GO:0006790 | sulfur compound metabolic process | 2,19E+00 | 5,25E+ 01 | 4,47E+ 02 | 6,24E+03 |
| 120 | GO:0006066 | alcohol metabolic process | 2,42E+00 | 5,73E+ 01 | 4,89E+ 02 | 6,88E+03 |
| 121 | GO:0010038 | response to metal ion | 3,07E+00 | 7,22E+ 01 | 6,16E+ 02 | 8,74E+03 |
| 122 | GO:0006105 | succinate metabolic process | 3,40E+00 | 7,92E+ 01 | 6,76E+ 02 | 9,67E+03 |

| 123 | GO:0019751 | polyol metabolic process | 3,44E+00 | 7,95E+ 01 | 6,78E+ 02 | 9,78E+03 |
| --- | --- | --- | --- | --- | --- | --- |
| 124 | GO:0010042 | response to manganese ion | 3,68E+00 | 8,44E+ 01 | 7,20E+ 02 | 1,05E+04 |
| 125 | GO:0051651 | maintenance of location in cell | 9,22E+00 | 2,10E+ 02 | 1,79E+ 03 | 2,63E+04 |
| 126 | GO:0042430 | indole-containing compound metabolic process | 9,65E+00 | 2,18E+ 02 | 1,86E+ 03 | 2,75E+04 |
| 127 | GO:0006090 | pyruvate metabolic process | 1,18E+01 | 2,64E+ 02 | 2,25E+ 03 | 3,35E+04 |
| 128 | GO:0043648 | dicarboxylic acid metabolic process | 1,21E+01 | 2,69E+ 02 | 2,29E+ 03 | 3,44E+04 |
| 129 | GO:0043603 | cellular amide metabolic process | 1,24E+01 | 2,75E+ 02 | 2,34E+ 03 | 3,54E+04 |
| 130 | GO:1903008 | organelle disassembly | 1,49E+01 | 3,25E+ 02 | 2,77E+ 03 | 4,23E+04 |
| 131 | GO:1902222 | erythrose 4-phosphate/phosphoenolpyruvate family amino acid catabolic process | 1,58E+01 | 3,41E+ 02 | 2,91E+ 03 | 4,50E+04 |
| 132 | GO:0006559 | L-phenylalanine catabolic process | 1,58E+01 | 3,41E+ 02 | 2,91E+ 03 | 4,50E+04 |

* Abbreviations: FDR = False Discovery Rate; B&H = Benjamini and Hochberg’s; B&Y = Benjamini–Yekutieli
