# Supplementary material for: Early Onset Ataxia with Comorbid Dystonia: Clinical, Anatomical and Biological Pathway Analysis Expose Shared Pathophysiology
Source: Diagnostics (Basel). 2020 Nov 24;10(12):997. doi: 10.3390/diagnostics10120997 (PMC7760948; doi:10.3390/diagnostics10120997)
Supplement: Supplementary file 1 [file diagnostics-10-00997-s001.zip › supplementary xml/11_Supplementary Table S11-xml.docx]

**Supplementary Table S11**. Common genes between networks EOA, AOA and dystonia.

| Common genes between networks EOA, AOA and dystonia | Common genes between networks EOA, dystonia | Common genes between networks AOA, dystonia |
| --- | --- | --- |
| *DPY30* | *PINK1* | *GINS1* |
| *ECHS1* | *CNBP* | *IMPDH2* |
| *PDHA1* | *ABCB7* | *SLC8A1* |
| *BYSL* | *PMPCA* | *DYNC1H1* |
| *PFKP* | *ATP5F1* | *SEZ6* |
| *ABCC6* | *MOSC1* | *PCBP3* |
| *DECR1* | *CUBN* | *PCBP1* |
| *TMEM151A* | *SLC25A3* | *ELAVL2* |
| *ACOT12* | *RAB1B* | *CALB2* |
| *CFHR5* | *LDB3* | *CHGB* |
| *SLC6A11* | *LILRB2* | *SBF1* |
| *GYS1* | *TRIB3* | *CYP4F2* |
| *TAF9B* | *NDUFAF4* | *AKAP6* |
| *RGN* | *NDUFAB1* | *PRPF8* |
| *HRG* | *COX6C* | *CHRNA3* |
| *NDUFS1* | *LYAR* | *CRYM* |
| *AATK* | *ATP5D* | *NUP93* |
| *PHYHIP* | *LONP1* | *RASL10B* |
| *GOT1* | *LEP* | *GPR22* |
| *PCSK1* | *PCYT2* | *SLC25A22* |
| *PTCD3* | *ECH1* | *ACHE* |
| *NAT10* | *DAD1* | *SYT1* |
| *LHFPL2* | *STX8* | *ELAVL4* |
| *DHRS11* | *CLTA* | *AZGP1* |
| *GRPEL1* | *ANLN* | *DDN* |
| *TBL3* | *KIAA0020* | *EIF2A* |
| *GPR37L1* | *TRA2B* | *RYR2* |
| *FGGY* | *LAMTOR2* | *APBA1* |
| *ALDOC* | *EVI2A* |  |
| *WDR45* | *TM4SF4* |  |
| *MGAT4B* | *C3* |  |
| *NEFH* | *COQ9* |  |
| *NPC2* | *NDUFA8* |  |
| *CABP1* | *COPS4* |  |
| *GJB1* | *PCBP4* |  |
| *ORM1* | *KL* |  |
| *C8G* | *NDUFS4* |  |
| *RBFOX1* | *TBC1D17* |  |
| *HPCA* | *GPD1* |  |
| *NAALADL1* | *NPM1* |  |
| *PSMD3* | *ERBB2IP* |  |
| *C9orf16* | *TF* |  |
| *FKRP* | *ALAD* |  |
| *ELAC2* | *POLG* |  |
| *DAO* | *CCT8* |  |
| *UQCRC1* | *SAMM50* |  |
| *TFR2* | *MRPL34* |  |
| *NCDN* | *MFN2* |  |
| *ENDOG* | *LDHB* |  |
| *AGT* | *THAP1* |  |
| *NACAD* | *IGSF11* |  |
| *CHI3L1* | *NDUFA2* |  |
| *COX10* | *POF1B* |  |
| *PCCB* | *ERO1L* |  |
| *CDHR5* | *SARS* |  |
| *LACTB2* | *QKI* |  |
| *ACO2* | *NCAM1* |  |
| *SLC39A12* | *CNTN2* |  |
| *HP* | *CDC6* |  |
| *GNAO1* | *SULT1C2* |  |
| *OPA1* | *DBH* |  |
| *POLD2* | *NBR1* |  |
| *MRPS28* | *NDUFC1* |  |
| *SLC35F1* | *MRPL15* |  |
| *KIAA0664* | *IMMT* |  |
| *BCKDK* | *UNG* |  |
| *CTSB* | *HDHD2* |  |
| *CAMK2B* | *AURKA* |  |
| *FETUB* | *SPG11* |  |
| *DCTN1* | *KCNJ13* |  |
| *C1QBP* | *AHCYL1* |  |
| *DNAJC11* | *GATA1* |  |
| *TM4SF20* | *DHFR* |  |
| *SEC13* | *PTPRH* |  |
| *ATP2B2* | *RP4-564F22.2* |  |
| *AQP8* | *FAM195A* |  |
| *KLHL21* | *GOT2* |  |
| *AKR1C4* | *ATP7B* |  |
| *SOX10* | *PROX1* |  |
| *EPS8L3* | *PPARGC1A* |  |
| *NUP85* | *ETFA* |  |
| *DPP7* | *38231* |  |
| *DGAT1* | *MTHFD1* |  |
| *ACADVL* | *ALDH6A1* |  |
| *ABCG8* | *SLC34A3* |  |
| *F2* | *SIRT2* |  |
| *AQP11* | *NDUFB8* |  |
| *APOA2* | *SMG8* |  |
| *GNS* | *NGFR* |  |
| *OLR1* | *DLST* |  |
| *LYPD3* | *NOP10* |  |
| *CYP2C9* | *PGM1* |  |
| *HNF4G* | *AURKAIP1* |  |
| *PRKDC* | *ATP5H* |  |
| *SLC17A4* | *GTF2H5* |  |
| *HPCAL4* | *HBP1* |  |
| *RRAGC* | *CA9* |  |
| *GPX4* | *SDHD* |  |
| *ITIH3* | *ARFGEF2* |  |
| *MCM5* | *SPG7* |  |
| *GAP43* | *VPS16* |  |
| *CPNE9* | *FOXRED1* |  |
| *MDH2* | *OXA1L* |  |
| *HN1* | *SIGLEC1* |  |
| *VEGFA* | *SLC9A2* |  |
| *NRSN1* | *ADD1* |  |
| *VAT1* | *S100B* |  |
| *PHYH* | *POP7* |  |
| *CPO* | *UGT2A3* |  |
| *MEP1A* | *ACLY* |  |
| *PARK2* | *GYPA* |  |
| *RUVBL2* | *CDK4* |  |
| *GPD1L* | *MRPS9* |  |
| *GPM6A* | *NDUFAF1* |  |
| *APOM* | *ZNF536* |  |
| *FARS2* | *UQCRQ* |  |
| *ADCYAP1R1* | *GAL3ST1* |  |
| *CA12* | *LAMA1* |  |
| *ALDH5A1* | *ENPP2* |  |
| *GABRA1* | *TUFM* |  |
| *BSG* | *MAN2C1* |  |
| *PCSK1N* | *MUS81* |  |
| *PROC* | *UQCC* |  |
| *SLCO1C1* | *PCSK2* |  |
| *SYP* | *AUH* |  |
| *FKBP8* | *SLC2A1* |  |
| *SYN2* | *GCDH* |  |
| *KIAA1109* | *SSR4* |  |
| *MIPEP* | *CKS2* |  |
| *FH* | *NDUFB3* |  |
| *VIL1* | *CCT7* |  |
| *COPS6* | *EEF1A2* |  |
| *HEBP1* | *CDK18* |  |
| *GRM3* | *LGALS2* |  |
| *PFAS* | *MCCC1* |  |
| *ATP5A1* | *SLC23A1* |  |
| *ACADM* | *CD302* |  |
| *MPZ* | *ATP5J2* |  |
| *SLC31A1* | *EIF2B4* |  |
| *PLA2G12B* | *ZFAND1* |  |
| *CTNS* | *SRP14* |  |
| *NEUROD2* | *RAD23A* |  |
| *CA4* | *COMMD1* |  |
| *GNG3* | *GHITM* |  |
| *ATP13A2* | *PRMT5* |  |
| *SCG2* | *PAX6* |  |
| *TREM2* | *FMO3* |  |
| *SLC17A6* | *COPE* |  |
| *SLC22A7* | *C8orf46* |  |
| *ACSBG1* | *SLC25A4* |  |
| *BNIP1* | *NDRG1* |  |
| *APOH* | *GBE1* |  |
| *C10orf2* | *PRIMA1* |  |
| *NDUFV1* | *WDR74* |  |
| *RBP2* | *FAM134A* |  |
| *SLC38A3* | *ATP5G3* |  |
| *TAGLN3* | *39692* |  |
| *MASP2* | *FHP1* |  |
| *DTYMK* | *ATP6V0C* |  |
| *F13B* | *C14orf79* |  |
| *KRT20* | *C2orf72* |  |
| *GABRD* | *IARS* |  |
| *UBE2O* | *DYNLL1* |  |
| *LINC00483* | *FEZ1* |  |
| *SLC25A11* | *UBL5* |  |
| *PVALB* | *APOD* |  |
| *ELOVL2* | *NELF* |  |
| *OSBPL1A* | *ATP5J* |  |
| *F10* | *PLEKHH1* |  |
| *APOO* | *C3orf26* |  |
| *CPNE6* | *ACSL1* |  |
| *SLC6A8* | *LRP2* |  |
| *CCT3* | *NDUFA1* |  |
| *NOP14* | *LIG1* |  |
| *MOBP* | *PMPCB* |  |
| *FBP1* | *MCM2* |  |
| *ADH4* | *CHST12* |  |
| *MCM4* | *MRPS36* |  |
| *SPTBN2* | *BZW2* |  |
| *CLPX* | *NSMCE1* |  |
| *CPLX2* | *NDUFA7* |  |
| *HADHB* | *GPR56* |  |
| *PTPRN* | *CHCHD10* |  |
| *KTI12* | *MRPL27* |  |
| *PDHX* | *NFS1* |  |
| *KNG1* | *CDC37* |  |
| *C21orf33* | *IDH3G* |  |
| *RPA3* | *ARFGAP3* |  |
| *PTGES2* | *RPUSD2* |  |
| *SCG3* | *DPEP1* |  |
| *GABRB3* | *CMTM5* |  |
| *PLG* | *GJC2* |  |
| *GDPD2* | *TRPM3* |  |
| *ATP6V1G2* | *P4HA1* |  |
| *PPA2* | *GPR17* |  |
| *NEUROD6* | *SLC7A7* |  |
| *LIPC* | *SFTPC* |  |
| *MBP* | *PSMC5* |  |
| *CPS1* | *ELOVL1* |  |
| *SLC2A2* | *RPS27L* |  |
| *ABCA2* | *UMOD* |  |
| *GABRA5* | *SRA1* |  |
| *METTL13* | *DIO1* |  |
| *MS4A12* | *CPOX* |  |
| *MAPK8IP3* | *CHADL* |  |
| *SRM* | *MAGOH* |  |
| *OTC* | *ERMN* |  |
| *TBRG4* | *NDRG4* |  |
| *AHSG* | *BNIP3L* |  |
| *RND2* | *ACACB* |  |
| *C19orf69* | *PITRM1* |  |
| *RGS17* | *EBNA1BP2* |  |
| *NUP205* | *CIAPIN1* |  |
| *G6PC* | *STAMBP* |  |
| *C17orf78* | *MRPS25* |  |
| *DYX1C1-CCPG1* | *TIMM50* |  |
| *AGXT* | *BOLA3* |  |
| *APOA4* | *NDUFB2* |  |
| *SH3GL2* | *MINOS1* |  |
| *KCNIP2* | *HARS2* |  |
| *U1* | *TPPP* |  |
| *PLP1* | *DNM1L* |  |
| *PEX12* | *MRPL23* |  |
| *RSL1D1* | *DHRS1* |  |
| *TPR* | *HIBADH* |  |
| *MTX2* | *NLE1* |  |
| *GLB1* | *C4BPA* |  |
| *CPN2* | *KIAA0564* |  |
| *STMN2* | *EIF3I* |  |
| *NFU1* | *KIF22* |  |
| *OMG* | *PHB* |  |
| *OLIG2* | *CKMT2* |  |
| *ANKRD43* | *HSDL2* |  |
| *MEP1B* | *CLTB* |  |
| *ANKRD34C* | *COX7C* |  |
| *ITIH4* | *NKX2-2* |  |
| *INSIG1* | *LIN28A* |  |
| *SERPINA10* | *CIDEB* |  |
| *SI* | *MECR* |  |
| *XPO7* | *CDC42EP1* |  |
| *APOC1* | *SERPINA3* |  |
| *STARD7* | *NDUFS3* |  |
| *MLYCD* | *WDR3* |  |
| *AVP* | *METTL17* |  |
| *SOAT2* | *MTCH2* |  |
| *SCN3B* | *TFF1* |  |
| *XPNPEP2* | *PPARGC1B* |  |
| *TMEM130* | *UGT8* |  |
| *FIBP* | *RNF123* |  |
| *QARS* | *FLII* |  |
| *ATP1A3* | *NARS* |  |
| *MRPS35* | *HRSP12* |  |
| *RBFOX3* | *PIPOX* |  |
| *C8A* | *FKBP2* |  |
| *SLC6A4* | *TMEM176B* |  |
| *NDUFAF3* | *LETMD1* |  |
| *SERPINA6* | *UQCRC2* |  |
| *GABARAP* | *MAEA* |  |
| *MBL2* | *UCK2* |  |
| *EIF3B* | *SLC44A4* |  |
| *FLVCR1* | *CLPB* |  |
| *RAB3C* | *COX5A* |  |
| *VSTM2L* | *AIFM1* |  |
| *SLC39A7* | *POLR2L* |  |
| *BCAN* | *MRPL4* |  |
| *AMBP* | *PCMTD2* |  |
| *TUBAL3* | *KLK6* |  |
| *CREB3L3* | *RLBP1* |  |
| *CLCN6* | *PNKP* |  |
| *ACAT1* | *PTRHD1* |  |
| *SYNGR3* | *LDHD* |  |
| *MDH1* | *KEL* |  |
| *LCT* | *SDHB* |  |
| *APOC3* | *JAM3* |  |
| *APEH* | *LAPTM4A* |  |
| *ANGPTL3* | *CA14* |  |
| *SLC25A1* | *CLDN11* |  |
| *VAMP1* | *ARL14* |  |
| *AFG3L2* | *RPP40* |  |
| *PON1* | *RPL22P22* |  |
| *GNPDA1* | *ATP5I* |  |
| *SLC26A3* | *ELOVL3* |  |
| *ACOX1* | *COQ7* |  |
| *HAO1* | *PAQR6* |  |
| *ATP5B* | *GLRX2* |  |
| *NEFM* | *MFAP3* |  |
| *GPI* | *TRIM31* |  |
| *COPZ1* | *NDUFS8* |  |
| *BCAP31* | *EIF2B3* |  |
| *VSNL1* | *FGG* |  |
| *C7orf42* | *ALDH4A1* |  |
| *ACP2* | *CRYAB* |  |
| *MOG* | *CKB* |  |
| *CHGA* | *MUC1* |  |
| *CACNA1B* | *TRMT112* |  |
| *CBR4* | *PKLR* |  |
| *SERPINA1* | *CD36* |  |
| *MAPT* | *HSD17B4* |  |
| *AC130454.2* | *IDH2* |  |
| *MYO16* | *XRCC6* |  |
| *MTTP* | *NOP2* |  |
| *GRSF1* | *NIPSNAP1* |  |
| *PHOX2B* | *CS* |  |
| *CPB2* | *FUT6* |  |
| *CC2D1A* | *FOLR1* |  |
| *NR2E1* | *GIP* |  |
| *POLA1* | *MAL* |  |
| *MCM3* | *GYPE* |  |
| *SV2B* | *GFI1B* |  |
| *NDUFS2* | *SCRG1* |  |
| *NHP2* | *PABPC3* |  |
| *SNAP25* | *KHK* |  |
| *FA2H* | *ESRRG* |  |
| *SLC13A2* | *APLP1* |  |
| *APOA5* | *ZCCHC24* |  |
| *MAPRE3* | *MS4A6A* |  |
| *AKR7A2* | *SLC34A1* |  |
| *GDAP1* | *OXCT1* |  |
| *RDBP* | *ABCA8* |  |
| *EPRS* | *NDUFA4L2* |  |
| *HMP19* | *CRYBA2* |  |
| *PDK2* | *ILK* |  |
| *CCDC86* | *FABP3* |  |
| *RUVBL1* | *MYBPC3* |  |
| *ARG1* | *MYOF* |  |
| *GFM1* | *CHL1* |  |
| *RPL7AL2* | *STC1* |  |
| *SULT2A1* | *MRPS33* |  |
| *HK1* | *CTD-3233P19.1* |  |
| *SLIRP* | *GSN* |  |
| *C10orf112* | *CPSF3* |  |
| *LRPPRC* | *BLM* |  |
| *ACO1* | *SNRPD1* |  |
| *ATP1B2* | *LGALS3BP* |  |
| *CDK5R2* | *ASPA* |  |
| *ECSIT* | *PCNA* |  |
| *HAPLN2* | *MMACHC* |  |
| *CALY* | *GPATCH4* |  |
| *CSE1L* | *CPN1* |  |
| *KCNJ10* | *CYC1* |  |
| *CTSD* | *SCD* |  |
| *SLC17A7* | *SCGN* |  |
| *FAM55D* | *FAM83E* |  |
| *PPT1* | *MAP6D1* |  |
| *SERPINF2* | *NDUFA4* |  |
| *GNB2* | *PCK1* |  |
| *SULT4A1* | *CYB561D2* |  |
| *SLC10A2* | *NDUFB5* |  |
| *FAM173A* | *CHCHD1* |  |
| *EXOSC5* | *AGPAT4* |  |
| *SERPIND1* | *NAA10* |  |
| *GDPD1* | *MRPL35* |  |
| *M6PR* | *CTA-714B7.6* |  |
| *OPALIN* | *GABARAPL1* |  |
| *ACADS* | *SLC4A2* |  |
| *ASGR1* | *KCNK1* |  |
| *EPHX2* | *CLCN7* |  |
| *CPT2* | *USP2* |  |
| *IVD* | *PPIB* |  |
| *HEATR1* | *COQ3* |  |
| *MTPAP* | *RBL2* |  |
| *TMPRSS15* | *VDAC1* |  |
| *IDH3A* | *TTC15* |  |
| *CA7* | *SCGB3A1* |  |
| *SUCLG1* | *FTSJ3* |  |
| *ACE* | *NDUFB6* |  |
| *L1CAM* | *TMEM27* |  |
| *MGAM* | *DQX1* |  |
| *ASGR2* | *PFKM* |  |
| *CBLN1* | *SEMA3B* |  |
| *SFRP5* | *MANEAL* |  |
| *GUCA2B* | *S100A1* |  |
| *ERP29* | *LHPP* |  |
| *TTR* | *AGXT2* |  |
| *BTNL8* | *PLK4* |  |
| *SCPEP1* | *MUC4* |  |
| *TFB2M* | *MCEE* |  |
| *MYO1A* | *DYNLRB1* |  |
| *LGMN* | *MRPS30* |  |
| *SERPINA7* | *ALDOA* |  |
| *INA* | *AIMP2* |  |
| *AKAP1* | *AC108134.2* |  |
| *CADM3* | *ZNF292* |  |
| *FABP1* | *PET112* |  |
| *TPP1* | *RPL36AL* |  |
| *C8B* | *HMGB2* |  |
| *CYP3A7* | *MRPL44* |  |
| *HSPA9* | *GAR1* |  |
| *MXI1* | *RP11-456A18.1* |  |
| *MAG* | *ACSM5* |  |
| *ORMDL2* | *ACSS2* |  |
| *IGFBP1* | *COX7A2P2* |  |
| *PLA2G15* | *RP4-581F12.1* |  |
| *GRIN1* | *KLF1* |  |
| *MYCBP2* | *HPD* |  |
| *SLC6A19* | *EEF2* |  |
| *CYP27A1* | *RFWD3* |  |
| *PUS7* | *SERGEF* |  |
| *SLC7A9* | *CLDN15* |  |
| *FAIM2* | *UROD* |  |
| *HADH* | *COMMD4* |  |
| *CACNG3* | *WDR11* |  |
| *CCL16* | *ATP5SL* |  |
| *LGI1* | *WDR46* |  |
| *SLC30A10* | *NSF* |  |
| *FMO5* | *GPR37* |  |
| *USP34* | *KCNJ1* |  |
| *PAQR5* | *NFASC* |  |
| *ITIH2* | *DDIT4* |  |
| *RPS17P5* | *PTGDS* |  |
| *CDKN2B-AS* | *RBP4* |  |
| *CARTPT* | *COX6B1* |  |
| *HNF4A* | *NOP56* |  |
| *DDC* | *JMJD6* |  |
| *SEMA4G* | *MCOLN1* |  |
| *CYP11B1* | *HIBCH* |  |
| *GPR172B* | *SORBS1* |  |
| *CEACAM7* | *ETFDH* |  |
| *RP3-486D24.1* | *POLR2K* |  |
| *SLC17A2* | *TRAPPC1* |  |
| *C15orf24* | *UCP1* |  |
| *MSH2* | *ERBB3* |  |
| *SLC12A5* | *MRPL39* |  |
| *BPHL* | *MUL1* |  |
| *MRPS2* | *PMP2* |  |
| *MAT1A* | *CREG1* |  |
| *LGI3* | *SLC12A1* |  |
| *CLYBL* | *BRP44L* |  |
| *LECT2* | *XPO1* |  |
| *PCCA* | *DCTPP1* |  |
| *ITFG1* | *SECISBP2L* |  |
| *ITIH1* | *LINC00461* |  |
| *SSSCA1* | *IFI30* |  |
| *NOX1* | *EEF1E1* |  |
| *HAAO* | *C19orf12* |  |
| *RAD51* | *HNRNPAB* |  |
| *RP1-101G11.2* | *FAR1* |  |
| *DLD* | *ABAT* |  |
| *CAMKV* | *ALDH1A1* |  |
| *S1PR5* | *PRR18* |  |
| *WDR18* | *TUBG1* |  |
| *ORM2* | *GBAS* |  |
| *EGLN3* | *BCAS1* |  |
| *HPX* | *SFTPB* |  |
| *CCL25* | *STAP2* |  |
| *XYLB* | *PARL* |  |
| *ADH6* | *ACAD10* |  |
| *COX4I1* | *SHFM1* |  |
| *F12* | *PTS* |  |
| *SETX* | *ALPI* |  |
| *C2orf28* | *FTSJ1* |  |
| *SERPINC1* | *ADSL* |  |
| *DLAT* | *FLAD1* |  |
| *TMEM48* | *TREH* |  |
| *FTCD* | *TMEM144* |  |
| *SCN2B* | *ABCG5* |  |
| *CA1* | *TSC22D4* |  |
| *AGMAT* | *ARL3* |  |
| *ESPN* | *COX7A1* |  |
| *RUNDC3A* | *COX5B* |  |
| *OGDHL* | *NEDD8* |  |
| *SNAP91* | *GFAP* |  |
| *NANS* | *C6orf136* |  |
| *NPC1* | *SUGP2* |  |
| *TUBB4A* | *AQP5* |  |
| *CYP3A4* | *RNASEH2A* |  |
| *TRAP1* | *FAM162A* |  |
| *GRIA2* | *AMN* |  |
| *PAH* | *C19orf60* |  |
| *ST18* | *PSMD13* |  |
| *SNCB* | *NDUFA3* |  |
| *CDHR2* | *IARS2* |  |
|  | *CHST5* |  |
|  | *ENPP6* |  |
|  | *FCN3* |  |
|  | *CISD1* |  |
|  | *FAM107A* |  |
|  | *NDUFA9* |  |
|  | *ADCK3* |  |
|  | *SERINC1* |  |
|  | *SLC25A12* |  |
|  | *CNP* |  |
|  | *MUC5AC* |  |
|  | *NNT* |  |
|  | *EIF3K* |  |
|  | *MRPL16* |  |
|  | *CNPPD1* |  |
|  | *ATP5C1* |  |
|  | *RARS* |  |
|  | *PSMC3* |  |
|  | *STMN4* |  |
|  | *AMT* |  |
|  | *MFSD5* |  |
|  | *TXN* |  |
|  | *FASTKD1* |  |
|  | *CFP* |  |
|  | *NR1I2* |  |
|  | *CTNNA2* |  |
|  | *NDUFA10* |  |
|  | *LARS2* |  |
|  | *TTPA* |  |
|  | *LPAR1* |  |
|  | *RELN* |  |
|  | *PPA1* |  |
|  | *ACOX2* |  |
|  | *LHX2* |  |
|  | *PDHB* |  |
|  | *UAP1* |  |
|  | *ECI1* |  |
|  | *DHODH* |  |
|  | *PRRT2* |  |
|  | *CUTA* |  |
|  | *CP* |  |
|  | *DUSP9* |  |
|  | *TM4SF5* |  |
|  | *SUCLA2* |  |
|  | *IDH3B* |  |
|  | *EIF3G* |  |
|  | *NDUFS7* |  |
|  | *ABCB8* |  |
|  | *PRMT7* |  |
|  | *USH1C* |  |
|  | *ADD2* |  |
|  | *SIN3B* |  |
|  | *CENPM* |  |
|  | *NDRG3* |  |
|  | *PEPD* |  |
|  | *GATA4* |  |
|  | *HHATL* |  |
|  | *PNKD* |  |
|  | *NDUFB9* |  |
|  | *ARNT2* |  |
|  |  |  |
