# Supplementary material for: Early Onset Ataxia with Comorbid Dystonia: Clinical, Anatomical and Biological Pathway Analysis Expose Shared Pathophysiology
Source: Diagnostics (Basel). 2020 Nov 24;10(12):997. doi: 10.3390/diagnostics10120997 (PMC7760948; doi:10.3390/diagnostics10120997)
Supplement: Supplementary file 1 [file diagnostics-10-00997-s001.zip › supplementary xml/12_Supplementary Table S12-xml.docx]

**Supplementary Table S12**. Top 10 GO Biological pathways common genes networks EAO and dystonia.

|  | **ID** | **Name** | **P Value** | **FDR* B&H** | **FDR* B&Y** | **Bonferroni** |
| --- | --- | --- | --- | --- | --- | --- |
| 1 | GO:0045333 | cellular respiration | 5,18E-37 | 2,95E-33 | 2,72E-32 | 2,95E-33 |
| 2 | GO:0017144 | drug metabolic process | 2,17E-36 | 6,19E-33 | 5,71E-32 | 1,24E-32 |
| 3 | GO:0015980 | energy derivation by oxidation of organic compounds | 1,80E-35 | 2,79E-32 | 2,57E-31 | 1,03E-31 |
| 4 | GO:0006091 | generation of precursor metabolites and energy | 1,96E-35 | 2,79E-32 | 2,57E-31 | 1,12E-31 |
| 5 | GO:0055114 | oxidation-reduction process | 2,03E-34 | 2,31E-31 | 2,14E-30 | 1,16E-30 |
| 6 | GO:0006754 | ATP biosynthetic process | 3,23E-33 | 3,07E-30 | 2,83E-29 | 1,84E-29 |
| 7 | GO:0009201 | ribonucleoside triphosphate biosynthetic process | 2,83E-32 | 2,17E-29 | 2,00E-28 | 1,61E-28 |
| 8 | GO:0022904 | respiratory electron transport chain | 3,05E-32 | 2,17E-29 | 2,00E-28 | 1,74E-28 |
| 9 | GO:0022900 | electron transport chain | 6,14E-32 | 3,88E-29 | 3,58E-28 | 3,49E-28 |
| 10 | GO:0009206 | purine ribonucleoside triphosphate biosynthetic process | 8,65E-32 | 4,44E-29 | 4,10E-28 | 4,93E-28 |

* Abbreviations: FDR = False Discovery Rate; B&H = Benjamini and Hochberg’s; B&Y= Benjamini–Yekutieli
