# Supplementary material for: Early Onset Ataxia with Comorbid Dystonia: Clinical, Anatomical and Biological Pathway Analysis Expose Shared Pathophysiology
Source: Diagnostics (Basel). 2020 Nov 24;10(12):997. doi: 10.3390/diagnostics10120997 (PMC7760948; doi:10.3390/diagnostics10120997)
Supplement: Supplementary file 1 [file diagnostics-10-00997-s001.zip › supplementary xml/13_Supplementary Table S13-xml.docx]

**Supplementary Table S13.** Top 10 GO Biological pathways common genes networks AOA and dystonia.

|  | **GO BP ID** | **Name of GO term** | **pValue** | **Bonferroni corrected pValue** |
| --- | --- | --- | --- | --- |
| 1 | GO:0010880 | regulation of release of sequestered calcium ion into cytosol by sarcoplasmic reticulum | 1,28E-02 | 1,63E+01 |
| 2 | GO:0014808 | release of sequestered calcium ion into cytosol by sarcoplasmic reticulum | 1,67E-02 | 2,13E+01 |
| 3 | GO:1903514 | release of sequestered calcium ion into cytosol by endoplasmic reticulum | 1,81E-02 | 2,31E+01 |
| 4 | GO:0098735 | positive regulation of the force of heart contraction | 1,86E-02 | 2,37E+01 |
| 5 | GO:0070296 | sarcoplasmic reticulum calcium ion transport | 2,86E-02 | 3,65E+01 |
| 6 | GO:0050804 | modulation of chemical synaptic transmission | 8,14E-02 | 1,04E+02 |
| 7 | GO:0099177 | regulation of trans-synaptic signaling | 8,22E-02 | 1,05E+02 |
| 8 | GO:0071313 | cellular response to caffeine | 1,02E-01 | 1,30E+02 |
| 9 | GO:0050773 | regulation of dendrite development | 1,34E-01 | 1,71E+02 |
| 10 | GO:0006937 | regulation of muscle contraction | 1,40E-01 | 1,78E+02 |
