# Supplementary material for: Early Onset Ataxia with Comorbid Dystonia: Clinical, Anatomical and Biological Pathway Analysis Expose Shared Pathophysiology
Source: Diagnostics (Basel). 2020 Nov 24;10(12):997. doi: 10.3390/diagnostics10120997 (PMC7760948; doi:10.3390/diagnostics10120997)
Supplement: Supplementary file 1 [file diagnostics-10-00997-s001.zip › supplementary xml/2. Supplementary Table S2-xml.docx]

**Supplementary Table 2.** Gene mutations vs MRI database.

| Ataxic genes | Disorder | comorbid dystonia (literature) | comorbid dystonia (UMCG) | Grey matter affected | White matter affected | Cerebellar Damage | Extracerebellar damage | Cerebellar Cortex | Cerebellar Nuclei | Cerebellar White matter | Vermis | Cerebellar (global) | Pons | Basal Ganglia | Thalamus | Cerebral white matter | spinal cord white matter | Spinal Cord | Brainstem | Cerebral cortex |
| --- | --- | --- | --- | --- | --- | --- | --- | --- | --- | --- | --- | --- | --- | --- | --- | --- | --- | --- | --- | --- |
| comorbid dystonia |  |  |  |  |  |  |  |  |  |  |  |  |  |  |  |  |  |  |  |  |
| *FXN* | FRDA | yes | yes | yes | yes | yes | yes |  |  | *A* |  | *A* |  |  | *D1* | *L* | *A* | *A* |  | *A* |
| *KIAA0586* | JBTS23 | yes | yes | yes | yes | yes | yes | *H* | *H* | *H* | *H* |  | *H* |  |  | *H* |  |  |  |  |
| *ATP1A3* | AHC2 | yes | yes | yes | yes | yes | yes | *A* |  |  |  | *A* |  | *D2* |  |  | *A* |  | *A* |  |
| *TTPA* | AVED | yes | yes | yes | yes | yes | yes | *A* |  | *A* |  | *A* | *A* |  |  |  |  | *A* |  |  |
| *CACNA1A* | SCA6 | yes | yes | yes | yes | yes | yes |  | *A* | *A* | *A* | *A* | *A* | *A* |  | *L* |  |  |  | *A* |
| *GOSR2* | EPM6 | yes | yes | yes | no | yes | yes |  |  |  | *A* | *A* |  |  |  |  |  |  |  | *A* |
| *SPTBN2* | SCA5 | yes | yes | yes | yes | yes | yes | *A* |  |  | *A* | *H* | *A* |  |  | *H* |  |  |  |  |
| *ATXN7* | SCA 7 | yes | yes | yes | yes | yes | no |  |  |  |  | *A* | *A* |  |  |  |  |  |  |  |
| *KCNC3* | SCA 13 | no | yes | yes | yes | yes | yes | *A* |  |  |  | *A* | *A* |  |  |  |  |  |  |  |
| *ATM* | AT | yes | yes | yes | yes | yes | yes | *A* |  |  | *A* | *A* |  |  |  | *L* |  |  |  |  |
| *CAMTA1* | CANPMR | no | yes | yes | no | yes | yes |  |  |  |  | *A* |  |  |  |  |  |  |  | *H* |
| *MT-ATP6* | NARP | yes | yes | yes | yes | yes | yes |  |  |  |  | *A* |  | *D3* |  | *L* |  |  |  | *A* |
| *ZMYND11* | MRD 30 | yes | yes | yes | yes | no | yes |  |  |  |  |  |  |  |  | *L* |  |  |  | *A* |
| *ALDH3A2* | SLS | yes | yes | yes | yes | no | yes |  |  |  |  |  |  |  |  | *L* |  |  |  | *A* |
| *TTF1* | BHC | yes | yes | no | no | no | no |  |  |  |  |  |  |  |  |  |  |  |  |  |
| *NPC1* | NPC | yes | yes | yes | yes | yes | yes |  |  |  |  | *A* | *A* | *A* | *A* | *L* | *A* |  |  | *A* |
| *LAMA1* | PTBHS | yes | yes | yes | yes | yes | yes |  |  |  | *H* |  |  |  |  | *L* |  |  |  |  |
| *CHD7* | CHARGE S | yes | yes | yes | no | yes | yes |  |  |  | *H* |  |  |  |  |  |  |  | *H* | *H* |
| *LYST* | CHS | yes | yes | yes | no | yes | yes |  |  |  |  | *A* |  |  |  | *L* |  |  |  | *A* |
| *HSD17B10* | MHBD-deficiency | yes | yes | yes | no | yes | yes |  |  |  |  | *A* |  |  |  |  |  |  |  | *A* |
| *EBF3* | HADDS | no | yes | yes | yes | yes | yes |  |  |  | *H* | *H* |  |  |  | *L* |  |  |  |  |
| *CTNNB1* | NEDSDV | yes | yes | yes | yes | no | yes |  |  |  |  |  |  |  |  | *L* |  |  | *H* | *H* |
| *HTT* | jHD | yes | yes | yes | yes | yes | yes |  |  | *H* |  | *A* |  | *A* |  | *L* |  |  |  | *A* |
| *SPG11* | SPG11 | yes | yes | yes | yes | yes | yes |  |  |  |  | *A* |  | *A* |  | *H* |  | *A* | *A* | *A* |
| *TUBB2A* | CDCBM5 | yes | yes | yes | yes | yes | yes |  |  |  | *H* | *A* | *H* |  |  | *L* |  |  | *H* | *H* |
|  |  |  |  |  |  |  |  |  |  |  |  |  |  |  |  |  |  |  |  |  |
| *Control group* |  |  |  |  |  |  |  |  |  |  |  |  |  |  |  |  |  |  |  |  |
| *KCND3* | SCA19 | yes | no | yes | no | yes | no |  |  |  | *A* | *A* |  |  |  |  |  |  |  |  |
| *ITPR1* | SCA29 | yes | no | yes | yes | yes | yes |  |  |  | *A* | *A* |  |  |  |  |  |  |  |  |
| *ABHD12* | PHARC | no | NE | yes | no | yes | yes |  |  |  |  | *A* |  |  |  |  |  |  |  | *A* |
| *IFRD1* | SCA18 | no | NE | yes | no | yes | no |  |  |  |  | *A* |  |  |  |  |  |  |  |  |
| *KIAA0226* | SCA15 | no | NE | yes | yes | yes | no |  |  |  |  | *A* |  |  |  |  |  |  |  |  |
| *PHYH* | Refsum disease | no | NE | yes | yes | no | yes |  |  |  |  |  |  |  |  | L |  |  | L | *A* |
| *TDP1* | SCAN1 | no | NE | yes | no | yes | yes |  |  |  |  | *A* |  |  |  |  |  |  |  | *A* |
| *VWA3B* | SCAR22 | no | NE | yes | yes | yes | yes |  |  |  |  | *A* |  |  |  | *A* |  |  |  |  |
| *GTF2H5* | TTD3 | no | NE | yes | yes | yes | yes |  |  |  |  | *A* |  |  |  | *L* |  |  |  | *A* |
| *FLVCR1* | AXPC1 | no | NE | yes | yes | no | yes |  |  |  |  |  |  |  |  |  | L |  |  |  |
| *ACO2* | ICRD | no | NE | yes | yes | yes | yes | *A* |  |  | *A* | *A* |  |  |  | *L* |  |  |  | *A* |
| *HSD17B4* | PRLTS1 | no | NE | yes | no | yes | no |  |  |  | *A* | *A* |  |  |  |  |  |  |  |  |
| *DNAJC3* | ACPHD | no | NE | yes | yes | yes | yes |  |  |  | *A* | *A* |  |  |  | *A* |  | *A* |  | *A* |

Legends: A, atrophy; H, hypoplasia; D, damage (D1, affected posterior thalamic radiations; D2, hypoxemic damage; D3, calcification); L, leukodystrophy (abnormal white matter); FRDA, Friedreich’s Ataxia; JBTS23, Joubert Syndrome 23; AHC, alternating hemiplegia of childhood2; AVED, Ataxia with isolated vitamin E deficiency; SCA#, spinocerebellar ataxia#; EPM6, progressive myoclonic epilepsy 6; AT, Ataxia Telangiectasia; CANPMR, cerebellar ataxia, nonprogressive with mental retardation; NARP, neuropathy ataxia and retinitis pigmentosa; MRD30, autosomal dominant mental retardation 30; SLS, sjogren-larsson syndrome; BHC, benign hereditary chorea; NPC, Niemann-Pick disease type C; PTBHS, Poretti-Boltshauser syndrome; CHARGE S, CHARGE syndrome; CHS, Chediak-Higashi syndrome; MHBD deficiency, HSD10 mitochondrial disease; HADDS, hypotonia ataxia and delayed development syndrome; NEDSDV, neurodevelopmental disorder with spastic diplegia and visual defect; jHD, juvenile Huntington Disease; SPG11, spastic paraplegia 11; CDCBM5, cortical dysplasia complex with other brain malformations 5; PHARC, polyneuropathy hearling loss ataxia retinitis pigmentosa and cataract; SCAN1, spinocerebellar ataxia with axonal neuropathy 1; SCAR22, spinocerebellar ataxia autosomal recessive 22; TTD3, trichothiodystrophy 3; AXPC1, ataxia posterior column with retinitis pigmentosa; ICRD, infantile cerebellar-retinal degeneration; PRLTS1, Perrault syndrome; ACPHD, ataxia combined cerebellar and peripheral with hearing loss and diabetes mellitus.
