# Supplementary material for: Early Onset Ataxia with Comorbid Dystonia: Clinical, Anatomical and Biological Pathway Analysis Expose Shared Pathophysiology
Source: Diagnostics (Basel). 2020 Nov 24;10(12):997. doi: 10.3390/diagnostics10120997 (PMC7760948; doi:10.3390/diagnostics10120997)
Supplement: Supplementary file 1 [file diagnostics-10-00997-s001.zip › supplementary xml/3. Supplementary Table S3-xml.docx]

**Supplementary Table S3.** Shared genes in ataxia and dystonia gene lists, obtained from gene panels, UMCG, Groningen, the Netherlands.

| **Genepanel EOA** | **Genepanel AOA** | **Genepanel Dystonia** |
| --- | --- | --- |
| *AARS2* | *ABHD12* | *ADAR* |
|  |  |  |
| *ABCB7* | *AFG3L2* | *ADAR* |
|  |  |  |
| *ABCD1* | *ANO10* | *ALDH5A1* |
|  |  |  |
| *ABHD12* | *APTX* | *ANO3* |
|  |  |  |
| *ACO2* | *ATCAY* | *ANO3* |
|  |  |  |
| *ACOX1* | *ATM* | *ANO3* |
|  |  |  |
| *AFG3L2* | *ATP1A3* | *ATP13A2* |
|  |  |  |
| *ALDH5A1* | *ATP2B3* | *ATP1A3* |
|  |  |  |
| *ANO10* | *CACNA1A* | *ATP7B* |
|  |  |  |
| *APTX* | *CACNA1G* | *BCS1L* |
|  |  |  |
| *ARHGEF2* | *CACNB4* | *C10orf2* |
|  |  |  |
| *ARSA* | *CAPN1* | *CACNA1B* |
|  |  |  |
| *ATCAY* | *CCDC88C* | *CDKL5* |
|  |  |  |
| *ATM* | *CLCN2* | *CIZ1* |
|  |  |  |
| *ATP1A3* | *CLN5* | *COX10* |
|  |  |  |
| *ATP2B3* | *COQ8A* | *COX15* |
|  |  |  |
| *ATP7A* | *CYP27A1* | *COX20* |
|  |  |  |
| *ATP7B* | *DNMT1* | *CP* |
|  |  |  |
| *ATP8A2* | *EEF2* | *DDC* |
|  |  |  |
| *AUH* | *ELOVL4* | *DLAT* |
|  |  |  |
| *BRAT1* | *ELOVL5* | *DLD* |
|  |  |  |
| *C19orf12* | *FAT2* | *FA2H* |
|  |  |  |
| *CA8* | *FGF14* | *FBXO7* |
|  |  |  |
| *CACNA1A* | *FLVCR1* | *FOLR1* |
|  |  |  |
| *CACNA2D2* | *FXN* | *FOXG1* |
|  |  |  |
| *CACNB4* | *GDAP2* | *FTL* |
|  |  |  |
| *CAMTA1* | *GFAP* | *FUS* |
|  |  |  |
| *CAPN1* | *GOSR2* | *GCDH* |
|  |  |  |
| *CLCN2* | *GRID2* | *GCH1* |
|  |  |  |
| *CLN3* | *GRM1* | *GNAL* |
|  |  |  |
| *CLN5* | *HEXA* | *KMT2B* |
|  |  |  |
| *CLP1* | *HEXB* | *LRPPRC* |
|  |  |  |
| *ADCK3* | *IFRD1* | *MECP2* |
|  |  |  |
| *CSTB* | *ITPR1* | *MTTP* |
|  |  |  |
| *CTBP1* | *KCNA1* | *NDUFA10* |
|  |  |  |
| *CWF19L1* | *KCNC3* | *NDUFA12* |
|  |  |  |
| *CYB5R3* | *KCND3* | *NDUFA2* |
|  |  |  |
| *CYP27A1* | *KIF26B* | *NDUFA9* |
|  |  |  |
| *DKC1* | *MRE11* | *NDUFAF2* |
|  |  |  |
| *DNAJC3* | *MTPAP* | *NDUFAF5* |
|  |  |  |
| *EBF3* | *MTTP* | *NDUFAF6* |
|  |  |  |
| *EIF2B2* | *NPC1* | *NDUFS1* |
|  |  |  |
| *EIF2B3* | *OPA1* | *NDUFS3* |
|  |  |  |
| *EIF2B4* | *PDYN* | *NDUFS4* |
|  |  |  |
| *EIF2B5* | *PEX10* | *NDUFS7* |
|  |  |  |
| *ERCC2* | *PHYH* | *NDUFS8* |
|  |  |  |
| *ERCC3* | *PIK3R5* | *NKX2-1* |
|  |  |  |
| *ERCC6* | *PLD3* | *NPC1* |
|  |  |  |
| *ERCC8* | *PMM2* | *NPC2* |
|  |  |  |
| *FA2H* | *PNKP* | *NUP62* |
|  |  |  |
| *FGF14* | *PNPLA6* | *PAH* |
|  |  |  |
| *FLVCR1* | *POLG* | *PANK2* |
|  |  |  |
| *FOLR1* | *PRKCG* | *PARK2* |
|  |  |  |
| *FXN* | *RNF170* | *PARK7* |
|  |  |  |
| *GALT* | *RNF216* | *PCBD1* |
|  |  |  |
| *GDAP2* | *RUBCN* | *PDHA1* |
|  |  |  |
| *GFAP* | *SACS* | *PDHB* |
|  |  |  |
| *GOSR2* | *SCN8A* | *PDHX* |
|  |  |  |
| *GPSM2* | *SETX* | *PINK1* |
|  |  |  |
| *GRID2* | *SIL1* | *PLA2G6* |
|  |  |  |
| *GRM1* | *SLC1A3* | *PLP1* |
|  |  |  |
| *GTF2H5* | *SLC2A1* | *PNKD* |
|  |  |  |
| *HEXA* | *SPG7* | *POLG* |
|  |  |  |
| *HEXB* | *SPTBN2* | *PRKRA* |
|  |  |  |
| *HSD17B4* | *STUB1* | *PRRT2* |
|  |  |  |
| *IFRD1* | *SYNE1* | *PTS* |
|  |  |  |
| *ITPR1* | *SYT14* | *QDPR* |
|  |  |  |
| *KCNA1* | *TDP1* | *RNASEH2A* |
|  |  |  |
| *KCNC3* | *TGM6* | *RNASEH2A* |
|  |  |  |
| *KCND3* | *TMEM240* | *RNASEH2C* |
|  |  |  |
| *KCNJ10* | *TPP1* | *SAMHD1* |
|  |  |  |
| *KIF1A* | *TRPC4* | *SCO2* |
|  |  |  |
| *KIF1C* | *TTBK2* | *SERAC1* |
|  |  |  |
| *L2HGDH* | *TTPA* | *SGCE* |
|  |  |  |
| *LAMA1* | *TUBB4A* | *SLC16A2* |
|  |  |  |
| *MCOLN1* | *TWNK* | *SLC19A3* |
|  |  |  |
| *MED17* | *VAMP1* | *SLC20A1* |
|  |  |  |
| *MED20* | *VWA3B* | *SLC2A1* |
|  |  |  |
| *MRE11A* | *WWOX* | *SLC30A10* |
|  |  |  |
| *MTPAP* | *ZNF592* | *SLC6A19* |
|  |  |  |
| *MTTP* |  | *SLC6A3* |
|  |  |  |
| *MVK* |  | *SPG11* |
|  |  |  |
| *NKX2-1* |  | *SPG7* |
|  |  |  |
| *NPC1* |  | *SPR* |
|  |  |  |
| *NPC2* |  | *SUCLA2* |
|  |  |  |
| *OPA1* |  | *SUCLG1* |
|  |  |  |
| *OPHN1* |  | *SURF1* |
|  |  |  |
| *PAX6* |  | *TACO1* |
|  |  |  |
| *PCNA* |  | *TAF1* |
|  |  |  |
| *PDHA1* |  | *TH* |
|  |  |  |
| *PEX10* |  | *THAP1* |
|  |  |  |
| *PEX7* |  | *TIMM8A* |
|  |  |  |
| *PHYH* |  | *TOR1A* |
|  |  |  |
| *PIGA* |  | *TREX1* |
|  |  |  |
| *PIGN* |  | *TTPA* |
|  |  |  |
| *PIK3R5* |  | *TUBB4A* |
|  |  |  |
| *PLA2G6* |  | *URI1* |
|  |  |  |
| *PLP1* |  | *VPS13A* |
|  |  |  |
| *PMM2* |  | *VPS13D* |
|  |  |  |
| *PMPCA* |  | *WDR45* |
|  |  |  |
| *PNKP* |  |  |
|  |  |  |
| *PNPLA6* |  |  |
|  |  |  |
| *POLG* |  |  |
|  |  |  |
| *POLR3A* |  |  |
|  |  |  |
| *POLR3B* |  |  |
|  |  |  |
| *PPT1* |  |  |
|  |  |  |
| *PRKCG* |  |  |
|  |  |  |
| *PRRT2* |  |  |
|  |  |  |
| *PSAP* |  |  |
|  |  |  |
| *PTF1A* |  |  |
|  |  |  |
| *QARS* |  |  |
|  |  |  |
| *RNF216* |  |  |
|  |  |  |
| *ROBO3* |  |  |
|  |  |  |
| *KIAA0226* |  |  |
|  |  |  |
| *SACS* |  |  |
|  |  |  |
| *SCN8A* |  |  |
|  |  |  |
| *SCYL1* |  |  |
|  |  |  |
| *SETX* |  |  |
|  |  |  |
| *SIL1* |  |  |
|  |  |  |
| *SLC17A5* |  |  |
|  |  |  |
| *SLC1A3* |  |  |
|  |  |  |
| *SLC2A1* |  |  |
|  |  |  |
| *SLC52A2* |  |  |
|  |  |  |
| *SLC9A1* |  |  |
|  |  |  |
| *SLC9A6* |  |  |
|  |  |  |
| *SNX14* |  |  |
|  |  |  |
| *SPG11* |  |  |
|  |  |  |
| *SPG7* |  |  |
|  |  |  |
| *SPTBN2* |  |  |
|  |  |  |
| *STUB1* |  |  |
|  |  |  |
| *STXBP1* |  |  |
|  |  |  |
| *SYNE1* |  |  |
|  |  |  |
| *SYNGAP1* |  |  |
|  |  |  |
| *TDP1* |  |  |
|  |  |  |
| *TDP2* |  |  |
|  |  |  |
| *TGM6* |  |  |
|  |  |  |
| *THG1L* |  |  |
|  |  |  |
| *TMEM240* |  |  |
|  |  |  |
| *TPP1* |  |  |
|  |  |  |
| *TTPA* |  |  |
|  |  |  |
| *TUBB4A* |  |  |
|  |  |  |
| *C10orf2* |  |  |
|  |  |  |
| *UBA5* |  |  |
|  |  |  |
| *VAMP1* |  |  |
|  |  |  |
| *VPS13D* |  |  |
|  |  |  |
| *VWA3B* |  |  |
|  |  |  |
| *KIAA0196* |  |  |
|  |  |  |
| *WDR73* |  |  |
|  |  |  |
| *WDR81* |  |  |
|  |  |  |
| *WFS1* |  |  |
|  |  |  |
| *WWOX* |  |  |
|  |  |  |
| *ZNF592* |  |  |
