# Supplementary material for: Early Onset Ataxia with Comorbid Dystonia: Clinical, Anatomical and Biological Pathway Analysis Expose Shared Pathophysiology
Source: Diagnostics (Basel). 2020 Nov 24;10(12):997. doi: 10.3390/diagnostics10120997 (PMC7760948; doi:10.3390/diagnostics10120997)
Supplement: Supplementary file 1 [file diagnostics-10-00997-s001.zip › supplementary xml/4. Supplementary Table S4-xml.docx]

**Supplementary Table S4a.** Scored Dystonic Comorbidity.

|  | Dystonia abs  observer 2 | Dystonia pres  observer 2 | TOTAL |
| --- | --- | --- | --- |
| Dystonia abs  observer 1 | 17 | 0 | 17 |
| Dystonia pres observer 1 | 11 | 52 | 63 |
| TOTAL | 28 | 52 | 80 |

Legends: recognition of comorbid dystonic features by observer 1 and 2. PRES = observed, ABS = not observed. In 11/80 patients, there was disagreement whether the dystonic features were really comorbid or rather physiologic (attributable to age).

**Supplementary Table S4b.**Comorbid Dystonia versus Disease Duration and Age of the Patients.

|  | Dystonia + | Dystonia - | *p-value^*^* |
| --- | --- | --- | --- |
| Disease duration in years (min – max) | 8.94 (0–28) | 7.65 (0–23) | *p = .645* |
| Age of the patient at video assessment in years (min – max) | 13.06 (1–36) | 15.71 (7–38) | *p = .103* |

Legends: + = present - = absent. The presence of comorbid dystonia is indicated by the recognition of abnormal dystonic features by both assessors. Disease duration and age of the patient are expressed as mean in years, min = minimal, max = maximal. There was no significant association between the presence of comorbid dystonia and disease duration or age.
