# Supplementary material for: Early Onset Ataxia with Comorbid Dystonia: Clinical, Anatomical and Biological Pathway Analysis Expose Shared Pathophysiology
Source: Diagnostics (Basel). 2020 Nov 24;10(12):997. doi: 10.3390/diagnostics10120997 (PMC7760948; doi:10.3390/diagnostics10120997)
Supplement: Supplementary file 1 [file diagnostics-10-00997-s001.zip › supplementary xml/5._Supplementary Table S5-xml.docx]

**Supplementary Table S5.** Frequency table of allocated damage on MRI.

|  | Comorbid Dystonia | | pValue* |
| --- | --- | --- | --- |
|  | yes | no |  |
| Cerebellar cortex | 6/25 (24.0%) | 1/13 (7.7%) | 0.385 |
| Cerebellar nuclei | 2/25 (8.0%) | 0/13 (0.0%) | 0.538 |
| Cerebellar white matter | 5/25 (20.0%) | 0/13 (0.0%) | 0.144 |
| Cerebellar vermis | 9/25 (36.0%) | 5/13 (38.5%) | 0.730 |
| Cerebellar (global) | 18/25 (72.0%) | 11/13 (84.6%) | 0.456 |
| Pons | 8/25 (32.0%) | 0/13 (0.0%) | **0.034** |
| Basal Ganglia | 6/25 (24.0%) | 0/13 (0.0%) | 0.076 |
| Thalamus | 2/25 (8.0%) | 0/13 (0.0%) | 0.538 |
| Cerebral White matter | 16/25 (64.0%) | 5/13 (38.5%) | 0.178 |
| Spinal cord White matter | 3/25 (12.0%) | 1/13 (7.7%) | 1.000 |
| Spinal cord | 3/25 (12.0%) | 1/13 (7.7%) | 1.000 |
| Brainstem | 5/25 (20.0%) | 1/13(7.7%) | 0.643 |
| Cerebral cortex | 15/25 (60.0%) | 6/13 (46.2%) | 0.502 |

Note: EOA phenotypes without comorbid dystonia did not reveal damage at the pons, basal ganglia, thalamus. Extra-cerebellar MRI damage at the pons and/or basal ganglia and/or thalamus *p < .001* (Fisher’s exact test). pValue in bold is statistically significant.
