# Supplementary material for: Early Onset Ataxia with Comorbid Dystonia: Clinical, Anatomical and Biological Pathway Analysis Expose Shared Pathophysiology
Source: Diagnostics (Basel). 2020 Nov 24;10(12):997. doi: 10.3390/diagnostics10120997 (PMC7760948; doi:10.3390/diagnostics10120997)
Supplement: Supplementary file 1 [file diagnostics-10-00997-s001.zip › supplementary xml/6. Supplementary Table S6-xml.docx]

**Supplementary Table S6.** Division of damage on MRI.

|  | Comorbid Dystonia |  |  |
| --- | --- | --- | --- |
|  | yes | no | pValue* |
| Grey matter damage  White matter damage  Cerebellar damage  Extracerebellar damage | 24/25 (96.0%)  19/25 (76.0%)  21/25 (84%)  23/25 (92.0%) | 13/13 (100%)  10/13 (76.9%)  11/13 (84.6%)  10/13 (76.9%) | 1.000  1.000  1.000  0.315 |

Note: Comorbid dystonia is neither associated with the division of MRI damage,
nor with grey- versus white- matter damage. * Fisher’s exact test used for calculating significance
