# Supplementary material for: Early Onset Ataxia with Comorbid Dystonia: Clinical, Anatomical and Biological Pathway Analysis Expose Shared Pathophysiology
Source: Diagnostics (Basel). 2020 Nov 24;10(12):997. doi: 10.3390/diagnostics10120997 (PMC7760948; doi:10.3390/diagnostics10120997)
Supplement: Supplementary file 1 [file diagnostics-10-00997-s001.zip › supplementary xml/7.Supplementary Table S7-xml.docx]

**Supplementary Table S7.** Shared genes between EOA, AOA and dystonia gene panels.

| **Shared genes EOA AOA** | **Shared genes EOA Dyst** | **Shared genes EAO AOA Dyst** |
| --- | --- | --- |
| *CACNA1A* | *C10orf2* | *TTPA* |
| *SETX* | *ATP7B* | *ATP1A3* |
| *TGM6* | *PDHA1* | *POLG* |
| *GOSR2* | *PLA2G6* | *NPC1* |
| *ZNF592* | *PLP1* | *TUBB4A* |
| *CYP27A1* | *FA2H* | *MTTP* |
| *APTX* | *PRRT2* | *SPG7* |
| *STUB1* | *FOLR1* | *SLC2A1* |
| *GRM1* | *NKX2-1* |  |
| *SLC1A3* | *VPS13D* |  |
| *CLCN2* | *ALDH5A1* |  |
| *PNKP* | *SPG11* |  |
| *PRKCG* | *NPC2* |  |
| *PEX10* |  |  |
| *SIL1* |  |  |
| *PMM2* |  |  |
| *ATCAY* |  |  |
| *KCND3* |  |  |
| *SACS* |  |  |
| *PHYH* |  |  |
| *CAPN1* |  |  |
| *FLVCR1* |  |  |
| *WWOX* |  |  |
| *CACNB4* |  |  |
| *HEXA* |  |  |
| *GRID2* |  |  |
| *ABHD12* |  |  |
| *SYNE1* |  |  |
| *GDAP2* |  |  |
| *PIK3R5* |  |  |
| *IFRD1* |  |  |
| *RNF216* |  |  |
| *TMEM240* |  |  |
| *SCN8A* |  |  |
| *KCNC3* |  |  |
| *SPTBN2* |  |  |
| *KCNA1* |  |  |
| *ITPR1* |  |  |
| *CLN5* |  |  |
| *FGF14* |  |  |
| *VWA3B* |  |  |
| *FXN* |  |  |
| *TPP1* |  |  |
| *PNPLA6* |  |  |
| *MTPAP* |  |  |
| *ANO10* |  |  |
| *HEXB* |  |  |
| *TDP1* |  |  |
| *VAMP1* |  |  |
| *ATM* |  |  |
| *AFG3L2* |  |  |
| *ATP2B3* |  |  |
| *GFAP* |  |  |
| *OPA1* |  |  |
