# Supplementary material for: Early Onset Ataxia with Comorbid Dystonia: Clinical, Anatomical and Biological Pathway Analysis Expose Shared Pathophysiology
Source: Diagnostics (Basel). 2020 Nov 24;10(12):997. doi: 10.3390/diagnostics10120997 (PMC7760948; doi:10.3390/diagnostics10120997)
Supplement: Supplementary file 1 [file diagnostics-10-00997-s001.zip › supplementary xml/8_Supplementary Table S8-xml.docx]

**Supplementary Table S8.** Enriched pathways EOA genes.

|  | **ID** | **Name** | **P Value** | **FDR* B&H** | **FDR* B&Y** | **Bonferroni** |
| --- | --- | --- | --- | --- | --- | --- |
| 1 | GO:0050905 | neuromuscular process | 4,91E-09 | 1,74E-05 | 1,52E-04 | 1,74E-05 |
| 2 | GO:0007626 | locomotory behavior | 4,76E-07 | 8,41E-04 | 7,36E-03 | 1,68E-03 |
| 3 | GO:0090659 | walking behavior | 1,38E-06 | 1,63E-03 | 1,43E-02 | 4,89E-03 |
| 4 | GO:0007610 | behavior | 4,76E-06 | 3,96E-03 | 3,46E-02 | 1,68E-02 |
| 5 | GO:0022008 | neurogenesis | 5,59E-06 | 3,96E-03 | 3,46E-02 | 1,98E-02 |
| 6 | GO:0008344 | adult locomotory behavior | 1,92E-05 | 1,13E-02 | 9,89E-02 | 6,78E-02 |
| 7 | GO:0042552 | myelination | 9,73E-05 | 3,74E-02 | 3,27E-01 | 3,44E-01 |
| 8 | GO:0006811 | ion transport | 9,99E-05 | 3,74E-02 | 3,27E-01 | 3,53E-01 |
| 9 | GO:0007272 | ensheathment of neurons | 1,15E-04 | 3,74E-02 | 3,27E-01 | 4,06E-01 |
| 10 | GO:0008366 | axon ensheathment | 1,15E-04 | 3,74E-02 | 3,27E-01 | 4,06E-01 |
| 11 | GO:0048878 | chemical homeostasis | 1,16E-04 | 3,74E-02 | 3,27E-01 | 4,11E-01 |
| 12 | GO:0009628 | response to abiotic stimulus | 2,59E-04 | 7,64E-02 | 6,68E-01 | 9,17E-01 |
| 13 | GO:0006629 | lipid metabolic process | 3,75E-04 | 1,02E-01 | 8,92E-01 | 1,33E+00 |
| 14 | GO:0030534 | adult behavior | 1,20E-03 | 2,83E-01 | 2,48E+00 | 4,24E+00 |
| 15 | GO:0007628 | adult walking behavior | 1,20E-03 | 2,83E-01 | 2,48E+00 | 4,25E+00 |
| 16 | GO:0034220 | ion transmembrane transport | 1,45E-03 | 3,21E-01 | 2,81E+00 | 5,14E+00 |
| 17 | GO:0055080 | cation homeostasis | 1,61E-03 | 3,35E-01 | 2,93E+00 | 5,69E+00 |
| 18 | GO:0014003 | oligodendrocyte development | 1,73E-03 | 3,40E-01 | 2,98E+00 | 6,12E+00 |
| 19 | GO:0098771 | inorganic ion homeostasis | 2,21E-03 | 4,11E-01 | 3,60E+00 | 7,82E+00 |
| 20 | GO:0050801 | ion homeostasis | 2,44E-03 | 4,12E-01 | 3,60E+00 | 8,62E+00 |
| 21 | GO:0030182 | neuron differentiation | 2,45E-03 | 4,12E-01 | 3,60E+00 | 8,65E+00 |
| 22 | GO:0050804 | modulation of chemical synaptic transmission | 2,83E-03 | 4,30E-01 | 3,76E+00 | 1,00E+01 |
| 23 | GO:0048699 | generation of neurons | 2,84E-03 | 4,30E-01 | 3,76E+00 | 1,01E+01 |
| 24 | GO:0099177 | regulation of trans-synaptic signaling | 2,92E-03 | 4,30E-01 | 3,76E+00 | 1,03E+01 |
| 25 | GO:0030003 | cellular cation homeostasis | 4,18E-03 | 5,87E-01 | 5,13E+00 | 1,48E+01 |
| 26 | GO:0048666 | neuron development | 4,31E-03 | 5,87E-01 | 5,13E+00 | 1,53E+01 |
| 27 | GO:0098660 | inorganic ion transmembrane transport | 5,57E-03 | 7,30E-01 | 6,38E+00 | 1,97E+01 |
| 28 | GO:0006873 | cellular ion homeostasis | 5,97E-03 | 7,54E-01 | 6,60E+00 | 2,11E+01 |
| 29 | GO:0098916 | anterograde trans-synaptic signaling | 7,58E-03 | 8,94E-01 | 7,82E+00 | 2,68E+01 |
| 30 | GO:0007268 | chemical synaptic transmission | 7,58E-03 | 8,94E-01 | 7,82E+00 | 2,68E+01 |
| 31 | GO:0055085 | transmembrane transport | 9,19E-03 | 1,02E+00 | 8,95E+00 | 3,25E+01 |
| 32 | GO:0099537 | trans-synaptic signaling | 9,25E-03 | 1,02E+00 | 8,95E+00 | 3,27E+01 |
| 33 | GO:0006812 | cation transport | 9,84E-03 | 1,05E+00 | 9,23E+00 | 3,48E+01 |
| 34 | GO:0048709 | oligodendrocyte differentiation | 1,10E-02 | 1,14E+00 | 9,99E+00 | 3,88E+01 |
| 35 | GO:0019752 | carboxylic acid metabolic process | 1,19E-02 | 1,19E+00 | 1,04E+01 | 4,23E+01 |
| 36 | GO:0099536 | synaptic signaling | 1,23E-02 | 1,19E+00 | 1,04E+01 | 4,35E+01 |
| 37 | GO:0007417 | central nervous system development | 1,24E-02 | 1,19E+00 | 1,04E+01 | 4,39E+01 |
| 38 | GO:0098655 | cation transmembrane transport | 1,47E-02 | 1,36E+00 | 1,19E+01 | 5,21E+01 |
| 39 | GO:0098662 | inorganic cation transmembrane transport | 1,50E-02 | 1,36E+00 | 1,19E+01 | 5,31E+01 |
| 40 | GO:0055082 | cellular chemical homeostasis | 1,63E-02 | 1,44E+00 | 1,26E+01 | 5,75E+01 |
| 41 | GO:0030001 | metal ion transport | 2,69E-02 | 2,32E+00 | 2,03E+01 | 9,51E+01 |
| 42 | GO:0048667 | cell morphogenesis involved in neuron differentiation | 3,41E-02 | 2,88E+00 | 2,52E+01 | 1,21E+02 |
| 43 | GO:0044255 | cellular lipid metabolic process | 4,01E-02 | 3,30E+00 | 2,89E+01 | 1,42E+02 |
| 44 | GO:0050885 | neuromuscular process controlling balance | 4,51E-02 | 3,62E+00 | 3,17E+01 | 1,59E+02 |
| 45 | GO:0043436 | oxoacid metabolic process | 6,32E-02 | 4,97E+00 | 4,35E+01 | 2,24E+02 |
| 46 | GO:0006082 | organic acid metabolic process | 8,73E-02 | 6,72E+00 | 5,88E+01 | 3,09E+02 |
| 47 | GO:0019725 | cellular homeostasis | 9,33E-02 | 7,02E+00 | 6,14E+01 | 3,30E+02 |
| 48 | GO:0055065 | metal ion homeostasis | 1,17E-01 | 8,60E+00 | 7,52E+01 | 4,13E+02 |
| 49 | GO:0006664 | glycolipid metabolic process | 1,41E-01 | 1,02E+01 | 8,91E+01 | 4,99E+02 |
| 50 | GO:0044242 | cellular lipid catabolic process | 1,50E-01 | 1,06E+01 | 9,24E+01 | 5,32E+02 |
| 51 | GO:1903509 | liposaccharide metabolic process | 1,52E-01 | 1,06E+01 | 9,24E+01 | 5,39E+02 |
| 52 | GO:0006820 | anion transport | 2,59E-01 | 1,76E+01 | 1,54E+02 | 9,16E+02 |
| 53 | GO:0016042 | lipid catabolic process | 2,76E-01 | 1,83E+01 | 1,60E+02 | 9,78E+02 |
| 54 | GO:0031175 | neuron projection development | 2,79E-01 | 1,83E+01 | 1,60E+02 | 9,87E+02 |
| 55 | GO:0061564 | axon development | 3,01E-01 | 1,94E+01 | 1,69E+02 | 1,06E+03 |
| 56 | GO:0006875 | cellular metal ion homeostasis | 3,72E-01 | 2,35E+01 | 2,06E+02 | 1,32E+03 |
| 57 | GO:0021782 | glial cell development | 4,73E-01 | 2,89E+01 | 2,53E+02 | 1,67E+03 |
| 58 | GO:0080171 | lytic vacuole organization | 4,88E-01 | 2,89E+01 | 2,53E+02 | 1,73E+03 |
| 59 | GO:0007040 | lysosome organization | 4,88E-01 | 2,89E+01 | 2,53E+02 | 1,73E+03 |
| 60 | GO:0006643 | membrane lipid metabolic process | 4,90E-01 | 2,89E+01 | 2,53E+02 | 1,73E+03 |
| 61 | GO:0001505 | regulation of neurotransmitter levels | 5,82E-01 | 3,35E+01 | 2,93E+02 | 2,06E+03 |
| 62 | GO:0015711 | organic anion transport | 5,87E-01 | 3,35E+01 | 2,93E+02 | 2,08E+03 |
| 63 | GO:0006687 | glycosphingolipid metabolic process | 6,02E-01 | 3,38E+01 | 2,96E+02 | 2,13E+03 |
| 64 | GO:0006681 | galactosylceramide metabolic process | 8,87E-01 | 4,90E+01 | 4,29E+02 | 3,14E+03 |
| 65 | GO:0019374 | galactolipid metabolic process | 1,32E+00 | 7,20E+01 | 6,30E+02 | 4,68E+03 |
| 66 | GO:0000904 | cell morphogenesis involved in differentiation | 1,68E+00 | 9,01E+01 | 7,89E+02 | 5,95E+03 |
| 67 | GO:0050877 | nervous system process | 1,91E+00 | 1,01E+02 | 8,80E+02 | 6,74E+03 |
| 68 | GO:0007409 | axonogenesis | 2,22E+00 | 1,16E+02 | 1,01E+03 | 7,86E+03 |
| 69 | GO:0048812 | neuron projection morphogenesis | 2,27E+00 | 1,17E+02 | 1,02E+03 | 8,05E+03 |
| 70 | GO:0072511 | divalent inorganic cation transport | 2,75E+00 | 1,39E+02 | 1,22E+03 | 9,73E+03 |
| 71 | GO:0070588 | calcium ion transmembrane transport | 2,82E+00 | 1,41E+02 | 1,23E+03 | 9,99E+03 |
| 72 | GO:0120039 | plasma membrane bounded cell projection morphogenesis | 3,04E+00 | 1,50E+02 | 1,31E+03 | 1,08E+04 |
| 73 | GO:0048858 | cell projection morphogenesis | 3,35E+00 | 1,62E+02 | 1,42E+03 | 1,18E+04 |
| 74 | GO:0006665 | sphingolipid metabolic process | 3,51E+00 | 1,68E+02 | 1,47E+03 | 1,24E+04 |
| 75 | GO:0010001 | glial cell differentiation | 3,74E+00 | 1,76E+02 | 1,54E+03 | 1,32E+04 |
| 76 | GO:0006281 | DNA repair | 4,16E+00 | 1,94E+02 | 1,69E+03 | 1,47E+04 |
| 77 | GO:0032990 | cell part morphogenesis | 4,86E+00 | 2,23E+02 | 1,95E+03 | 1,72E+04 |
| 78 | GO:0009314 | response to radiation | 4,99E+00 | 2,27E+02 | 1,98E+03 | 1,77E+04 |
| 79 | GO:0021695 | cerebellar cortex development | 6,22E+00 | 2,79E+02 | 2,44E+03 | 2,20E+04 |
| 80 | GO:0035637 | multicellular organismal signaling | 6,32E+00 | 2,80E+02 | 2,45E+03 | 2,24E+04 |
| 81 | GO:0006672 | ceramide metabolic process | 6,45E+00 | 2,82E+02 | 2,46E+03 | 2,28E+04 |
| 82 | GO:0048732 | gland development | 6,62E+00 | 2,85E+02 | 2,49E+03 | 2,34E+04 |
| 83 | GO:0006836 | neurotransmitter transport | 6,68E+00 | 2,85E+02 | 2,49E+03 | 2,36E+04 |
| 84 | GO:0060322 | head development | 7,72E+00 | 3,25E+02 | 2,84E+03 | 2,73E+04 |
| 85 | GO:0050884 | neuromuscular process controlling posture | 7,90E+00 | 3,29E+02 | 2,88E+03 | 2,79E+04 |
| 86 | GO:0007420 | brain development | 1,11E+01 | 4,57E+02 | 4,00E+03 | 3,93E+04 |
| 87 | GO:0042063 | gliogenesis | 1,17E+01 | 4,74E+02 | 4,15E+03 | 4,13E+04 |
| 88 | GO:0021696 | cerebellar cortex morphogenesis | 1,21E+01 | 4,87E+02 | 4,26E+03 | 4,28E+04 |
| 89 | GO:0006677 | glycosylceramide metabolic process | 1,24E+01 | 4,92E+02 | 4,30E+03 | 4,37E+04 |
| 90 | GO:0043574 | peroxisomal transport | 1,26E+01 | 4,97E+02 | 4,35E+03 | 4,47E+04 |

* Abbreviations: FDR = False Discovery Rate; B&H = Benjamini and Hochberg’s; B&Y= Benjamini–Yekutieli
