# Supplementary material for: Early Onset Ataxia with Comorbid Dystonia: Clinical, Anatomical and Biological Pathway Analysis Expose Shared Pathophysiology
Source: Diagnostics (Basel). 2020 Nov 24;10(12):997. doi: 10.3390/diagnostics10120997 (PMC7760948; doi:10.3390/diagnostics10120997)
Supplement: Supplementary file 1 [file diagnostics-10-00997-s001.zip › supplementary xml/9_Supplementary Table S9-xml.docx]

**Supplementary Table S9.** Enriched pathways AOA genes.

|  | **ID** | **Name** | **P Value** | **FDR B&H*** | **FDR B&Y*** | **Bonferroni** |
| --- | --- | --- | --- | --- | --- | --- |
| 1 | GO:0050905 | neuromuscular process | 1,58E-04 | 3,63E-01 | 3,04E+00 | 3,92E-01 |
| 2 | GO:0008344 | adult locomotory behavior | 3,32E-04 | 3,63E-01 | 3,04E+00 | 8,25E-01 |
| 3 | GO:0007628 | adult walking behavior | 4,37E-04 | 3,63E-01 | 3,04E+00 | 1,09E+00 |
| 4 | GO:0090659 | walking behavior | 6,02E-04 | 3,75E-01 | 3,15E+00 | 1,50E+00 |
| 5 | GO:0030534 | adult behavior | 2,93E-03 | 1,46E+00 | 1,22E+01 | 7,29E+00 |
| 6 | GO:0007626 | locomotory behavior | 4,20E-03 | 1,74E+00 | 1,46E+01 | 1,04E+01 |
| 7 | GO:0098660 | inorganic ion transmembrane transport | 2,84E-02 | 8,83E+00 | 7,41E+01 | 7,05E+01 |
| 8 | GO:0034220 | ion transmembrane transport | 2,84E-02 | 8,83E+00 | 7,41E+01 | 7,06E+01 |
| 9 | GO:0098916 | anterograde trans-synaptic signaling | 4,92E-02 | 1,20E+01 | 1,01E+02 | 1,22E+02 |
| 10 | GO:0007268 | chemical synaptic transmission | 4,92E-02 | 1,20E+01 | 1,01E+02 | 1,22E+02 |
| 11 | GO:0099537 | trans-synaptic signaling | 5,68E-02 | 1,20E+01 | 1,01E+02 | 1,41E+02 |
| 12 | GO:0098655 | cation transmembrane transport | 5,78E-02 | 1,20E+01 | 1,01E+02 | 1,44E+02 |
| 13 | GO:0099536 | synaptic signaling | 6,99E-02 | 1,25E+01 | 1,05E+02 | 1,74E+02 |
| 14 | GO:0055085 | transmembrane transport | 7,06E-02 | 1,25E+01 | 1,05E+02 | 1,76E+02 |
| 15 | GO:0055082 | cellular chemical homeostasis | 8,57E-02 | 1,42E+01 | 1,19E+02 | 2,13E+02 |
| 16 | GO:0048878 | chemical homeostasis | 1,40E-01 | 2,17E+01 | 1,83E+02 | 3,48E+02 |
| 17 | GO:0035637 | multicellular organismal signaling | 2,32E-01 | 3,20E+01 | 2,69E+02 | 5,76E+02 |
| 18 | GO:0098662 | inorganic cation transmembrane transport | 2,32E-01 | 3,20E+01 | 2,69E+02 | 5,77E+02 |
| 19 | GO:0006811 | ion transport | 2,46E-01 | 3,22E+01 | 2,71E+02 | 6,12E+02 |
| 20 | GO:0030001 | metal ion transport | 3,48E-01 | 4,33E+01 | 3,63E+02 | 8,65E+02 |
| 21 | GO:0006812 | cation transport | 3,95E-01 | 4,68E+01 | 3,93E+02 | 9,82E+02 |
| 22 | GO:0055080 | cation homeostasis | 6,28E-01 | 7,10E+01 | 5,96E+02 | 1,56E+03 |
| 23 | GO:0098771 | inorganic ion homeostasis | 7,64E-01 | 8,26E+01 | 6,94E+02 | 1,90E+03 |
| 24 | GO:0019725 | cellular homeostasis | 8,32E-01 | 8,62E+01 | 7,24E+02 | 2,07E+03 |
| 25 | GO:0030003 | cellular cation homeostasis | 8,90E-01 | 8,85E+01 | 7,43E+02 | 2,21E+03 |
| 26 | GO:0007610 | behavior | 9,64E-01 | 9,22E+01 | 7,74E+02 | 2,40E+03 |
| 27 | GO:0006873 | cellular ion homeostasis | 1,11E+00 | 1,02E+02 | 8,60E+02 | 2,77E+03 |
| 28 | GO:0050804 | modulation of chemical synaptic transmission | 1,19E+00 | 1,04E+02 | 8,77E+02 | 2,97E+03 |
| 29 | GO:0099177 | regulation of trans-synaptic signaling | 1,22E+00 | 1,04E+02 | 8,77E+02 | 3,03E+03 |
| 30 | GO:0070588 | calcium ion transmembrane transport | 1,49E+00 | 1,23E+02 | 1,03E+03 | 3,69E+03 |
| 31 | GO:0050801 | ion homeostasis | 2,10E+00 | 1,69E+02 | 1,42E+03 | 5,23E+03 |
| 32 | GO:0006629 | lipid metabolic process | 2,91E+00 | 2,26E+02 | 1,90E+03 | 7,23E+03 |
| 33 | GO:0042391 | regulation of membrane potential | 5,34E+00 | 4,03E+02 | 3,38E+03 | 1,33E+04 |
| 34 | GO:0055065 | metal ion homeostasis | 5,71E+00 | 4,17E+02 | 3,50E+03 | 1,42E+04 |
| 35 | GO:0006836 | neurotransmitter transport | 6,16E+00 | 4,38E+02 | 3,67E+03 | 1,53E+04 |
| 36 | GO:0050885 | neuromuscular process controlling balance | 8,09E+00 | 5,59E+02 | 4,69E+03 | 2,01E+04 |
| 37 | GO:0007267 | cell-cell signaling | 8,97E+00 | 6,03E+02 | 5,06E+03 | 2,23E+04 |
| 38 | GO:0006875 | cellular metal ion homeostasis | 9,23E+00 | 6,04E+02 | 5,07E+03 | 2,29E+04 |
| 39 | GO:0098506 | polynucleotide 3' dephosphorylation | 1,52E+01 | 9,72E+02 | 8,16E+03 | 3,79E+04 |

* Abbreviations: FDR = False Discovery Rate; B&H = Benjamini and Hochberg’s; B&Y= Benjamini–Yekutieli
