# Supplementary material for: Early Onset Ataxia with Comorbid Dystonia: Clinical, Anatomical and Biological Pathway Analysis Expose Shared Pathophysiology
Source: Diagnostics (Basel). 2020 Nov 24;10(12):997. doi: 10.3390/diagnostics10120997 (PMC7760948; doi:10.3390/diagnostics10120997)
Supplement: Supplementary file 1 [file diagnostics-10-00997-s001.zip › supplementary xml/Supplementary Figures S1-5-xml.docx]

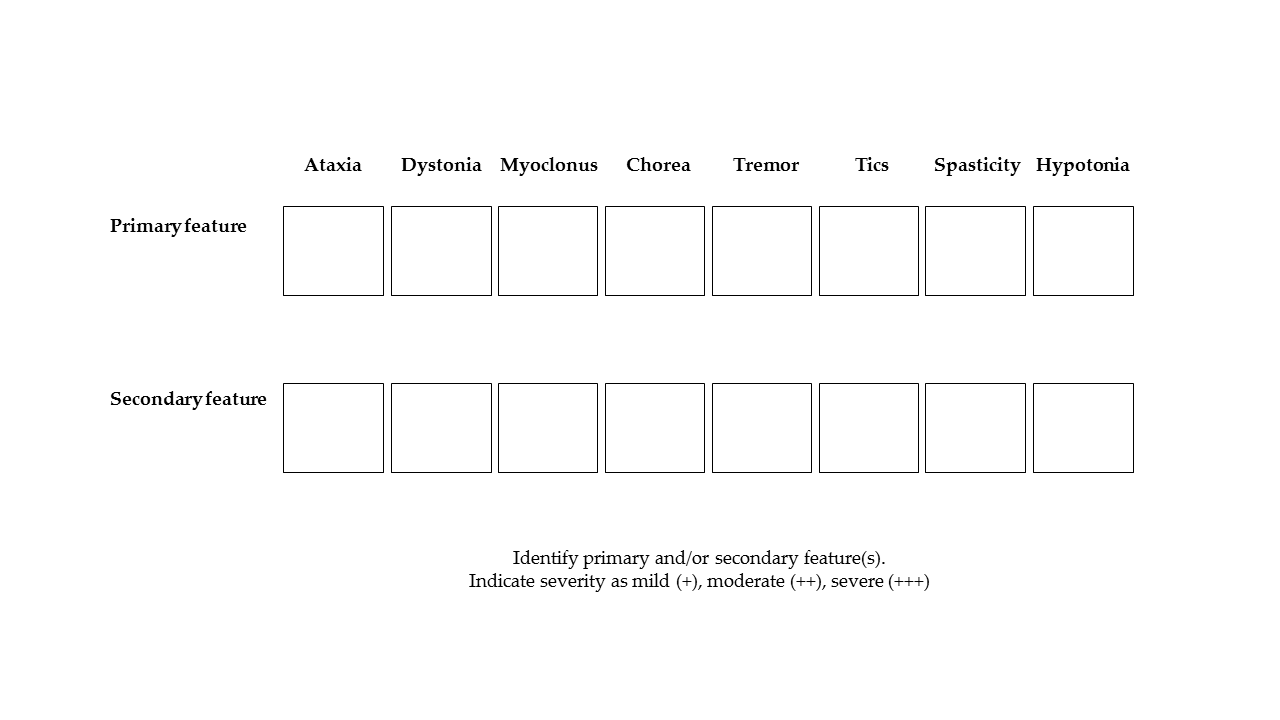


**Supplementary Figure S1.** Phenotypic Assessment Form. Modified from Lawerman T.F. et al [1].


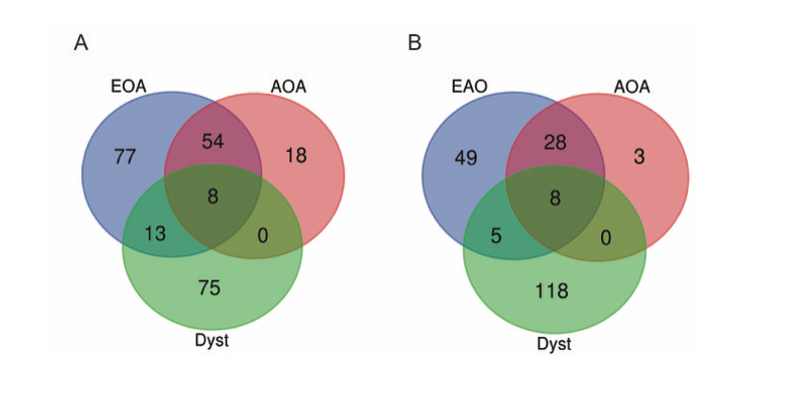


**Supplementary Figure S2:** Legend **(A)** Venn diagram plot comparing genes between EOA (N=152 genes), AOA (N=80 genes) and dystonia (N=100 genes). Eight common genes are identified between EOA, AOA and dystonia.(**B)** Venn diagram plot comparing GO Biological Pathways between EOA (N=90 pathways), AOA (N=39 pathways) and dystonia (N=131 pathways). Eight common biological pathwatys are identified between EOA, AOA and dystonia.


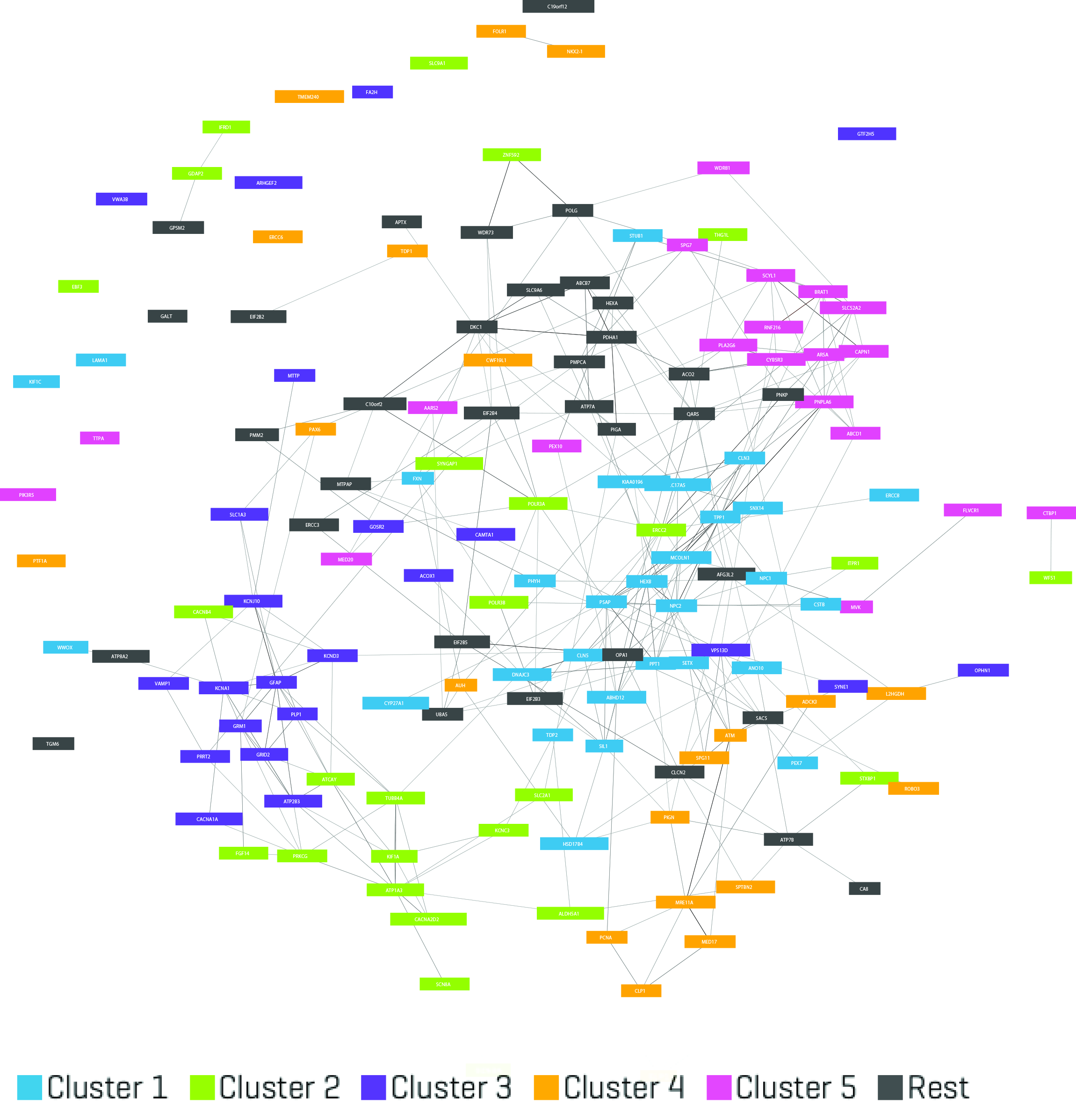


**Supplementary Figure S3.** Schematic representation of network of EOA genes comprised of 7 clusters generated by GeneNetwork (GeneNetwork.nl).

**Supplementary Figure S4.** Schematic representation of network of AOA genes comprised of three clusters generated by GeneNetwork (GeneNetwork.nl).

**Supplementary Figure S5.** Schematic representation of network of dystonia genes comprised of four clusters generated by GeneNetwork (GeneNetwork.nl).
